# Co-occurring clonal hematopoiesis exhibits strong selection and high leukemia risk

**DOI:** 10.1038/s41467-026-73302-x

**Published:** 2026-05-21

**Authors:** Kara M. Barnao, Aubrey K. Hubbard, Irenaeus C. C. Chan, Weiyin Zhou, Yasminka A. Jakubek, Giulio Genovese, Wendy S. W. Wong, Rebecca L. Kelly, Corey D. Young, Derek W. Brown, Wen-Yi Huang, Neal D. Freedman, Kristine Jones, Amy Hutchinson, Belynda Hicks, Duc Tran, Donna Arnett, Kathleen C. Barnes, Joshua C. Bis, Eric Boerwinkle, Jennifer A. Brody, April P. Carson, Daniel I. Chasman, Michael H. Cho, Pinkal Desai, Margaret F. Doyle, Myriam Fornage, Xiuqing Guo, Nancy Heard-Costa, Marguerite Ryan Irvin, Andrew D. Johnson, Sharon L. R. Kardia, Charles Kooperberg, Daniel Levy, Joshua P. Lewis, Yun Li, Ruth J. F. Loos, Taralynn M. Mack, Rasika A. Mathias, Braxton D. Mitchell, Kari E. North, Nathan Pankratz, Patricia A. Peyser, Michael H. Preuss, Bruce M. Psaty, Laura M. Raffield, Susan Redline, Stephen S. Rich, Jerome I. Rotter, Edwin K. Silverman, Albert V. Smith, Jennifer A. Smith, Adrienne Stilp, Yin Cao, Paul Scheet, Alexander P. Reiner, Alexander G. Bick, Stephen J. Chanock, Paul L. Auer, Kelly L. Bolton, Mitchell J. Machiela

**Affiliations:** 1 Division of Cancer Epidemiology and Genetics, National Cancer Institute, NIH, Rockville, MD USA; 2 Division of Oncology, Department of Medicine, Washington University School of Medicine, St. Louis, MO USA; 3 Cancer Genomics Research Laboratory, Frederick National Laboratory for Cancer Research, Leidos Biomedical Research Inc., Frederick, MD USA; 4 Department of Internal Medicine, University of Kentucky, Lexington, KY USA; 5 Program in Medical and Population Genetics, Broad Institute of MIT and Harvard, Cambridge, MA USA; 6 University of South Carolina, Columbia, SC USA; 7 Galatea Bio, Inc., Miami, FL USA; 8 Cardiovascular Health Research Unit, Department of Medicine, University of Washington, Seattle, WA USA; 9 Department of Epidemiology, Human Genetics, and Environmental Sciences, Human Genetics Center, School of Public Health, The University of Texas Health Science Center at Houston, Houston, TX USA; 10 Department of Medicine, University of Mississippi Medical Center, Jackson, MS USA; 11 Harvard Medical School, Boston, MA USA; 12 Division of Preventative Medicine, Brigham and Women’s Hospital, Boston, MA USA; 13 Channing Division of Network Medicine, Brigham and Women’s Hospital, Boston, MA USA; 14 Division of Hematology and Oncology, Weill Cornell Medical College, New York, NY USA; 15 Department of Pathology and Laboratory Medicine, The University of Vermont Lerner College of Medicine, Colchester, VT USA; 16 Institute of Molecular Medicine, McGovern Medical School, The University of Texas Health Science Center at Houston, Houston, TX USA; 17 Department of Medicine, The Institute for Translational Genomics and Population Sciences, The Lundquist Institute for Biomedical Innovation at Harbor-UCLA Medical Center, Torrance, CA USA; 18 Department of Medicine, Boston University School of Medicine, Boston, MA USA; 19 Department of Epidemiology, University of Alabama at Birmingham, Birmingham, AL USA; 20 Population Sciences Branch, Division of Intramural Research, National Heart, Lung, and Blood Institute, National Institutes of Health, Bethesda, MD USA; 21 Department of Epidemiology, School of Public Health, University of Michigan, Ann Arbor, MI USA; 22 Division of Public Health Science, Fred Hutchinson Cancer Center, Seattle, WA USA; 23 Department of Medicine, University of Maryland Baltimore, Baltimore, MD USA; 24 Department of Genetics, Department of Biostatistics, University of North Carolina at Chapel Hill, Chapel Hill, NC USA; 25 The Charles Bronfman Institute for Personalized Medicine, Icahn School of Medicine at Mount Sinai, New York, NY USA; 26 Novo Nordisk Foundation Center for Basic Metabolic Research, Faculty of Health and Medical Sciences, University of Copenhagen, Copenhagen, Denmark; 27 Division of Genetic Medicine, Vanderbilt University Medical Center, Nashville, TN USA; 28 Laboratory of Allergic Diseases, National Institute of Allergy and Infectious Diseases, NIH, Bethesda, MD USA; 29 Department of Epidemiology, University of North Carolina at Chapel Hill, Chapel Hill, NC USA; 30 Depart of Laboratory Medicine and Pathology, University of Minnesota, Minneapolis, MN USA; 31 Department of Medicine, University of Washington, Seattle, WA USA; 32 Department of Health Systems and Population Health, University of Washington, Seattle, WA USA; 33 Department of Genetics, University of North Carolina at Chapel Hill, Chapel Hill, NC USA; 34 Division of Sleep Medicine, Harvard Medical School, Boston, MA USA; 35 Department of Genome Sciences, University of Virginia School of Medicine, Charlottesville, VA USA; 36 The Institute for Translational Genomics and Population Sciences, Department of Pediatrics, The Lundquist Institute for Biomedical Innovation at Harbor-UCLA Medical Center, Torrance, CA USA; 37 Department of Biostatistics and Center for Statistical Genetics, University of Michigan, Ann Arbor, MI USA; 38 Survey Research Center, Institute for Social Research, University of Michigan, Ann Arbor, MI USA; 39 Department of Biostatistics, University of Washington, Seattle, WA USA; 40 Division of Public Health Sciences, Department of Surgery, Washington University School of Medicine, St. Louis, MO USA; 41 Alvin J. Siteman Cancer Center, Washington University School of Medicine, St. Louis, MO USA; 42 Division of Gastroenterology, Department of Medicine, Washington University School of Medicine, St. Louis, MO USA; 43 Department of Epidemiology, University of Texas MD Anderson Cancer Center, Houston, TX USA; 44 Department of Epidemiology, University of Washington, Seattle, WA USA; 45 Division of Biostatistics, Data Science Institute, and Cancer Center, Medical College of Wisconsin, Milwaukee, WI USA

**Keywords:** Haematological cancer, Cancer epidemiology, Cancer genetics

## Abstract

Clonal hematopoiesis of indeterminate potential (CHIP) and mosaic chromosomal alterations (mCAs) are two types of clonal hematopoiesis (CH) associated with hematological parameters and malignancy risk. Here we show, in genomic data from 546,090 biobank participants, that co-occurring CH (≥2 CH mutations detected) is present in 1.6% of cancer-free individuals and shows strong evidence for selection (up to 804x enrichment). Co-occurrence is more frequent in those with a prior cancer (3.6%), suggesting treatment-induced selection. Acquisition of CHIP usually precedes mCAs with co-occurrences manifesting stronger phenotypic disruptions in telomere attrition and hematologic parameters than component CH events. Individuals with co-occurring CH have pronounced elevations in risk of myeloid and lymphoid malignancies (HRs>40), particularly when CHIP and mCAs overlap genomically. Our findings indicate CH co-occurrences are selected for in the aging population and identify CH clones with notable implications for future malignancy risk.

## Abstract (web summary)

Results from integrative population-based investigations indicate co-occurring types of clonal hematopoiesis are highly enriched and markedly increase blood cancer risk, highlighting new opportunities for early detection and targeted surveillance of high-risk individuals.

## Introduction

The accumulation of post-zygotic mutations from environmental exposures and DNA replication errors provides the foundation for molecular evolution. While most somatic mutations are inconsequential, some promote dysregulation of core cellular pathways, leading to the acquisition or selection of additional mutations favoring progression from premalignant states to cancer^1^. Tissue-specific knowledge of commonly acquired mutations, their co-occurrence, and interactions can provide novel etiologic insights critical for identifying precancerous clones with high potential for malignant transformation.

Clonal hematopoiesis (CH) is the expansion of hematopoietic stem and progenitor cells containing post-zygotic mutations in individuals with normal hematologic parameters. Clonal proliferation can be driven by single-nucleotide variants (SNVs) or small insertions and deletions (indels) in leukemia driver genes, termed clonal hematopoiesis of indeterminate potential (CHIP). Previous studies have observed *DNMT3A, TET2, ASXL1, PPM1D*, and *JAK2* as the most frequent genes mutated in CHIP^2–6^. CH can also be driven by large (typically megabase-scale) structural chromosomal changes (e.g., chromosomal gains, losses, or copy-neutral loss of heterozygosity, CNLOH), collectively termed mosaic chromosomal alterations (mCAs). The most frequently detected mCAs are loss of the sex chromosomes: mosaic loss of the Y chromosome (mLOY) in males and mosaic loss of the X chromosome (mLOX) in females. CH has increased population frequencies as age advances, demonstrating the erosive associations of aging on genomic integrity.

A sizeable fraction of cancer-free individuals have some type of detectable CH that varies in frequency based on population characteristics (e.g., estimated population frequencies CHIP = 6%^7^, autosomal mCAs = 3.5%^8^, mLOY = 20%^9^, and mLOX = 12%^10^). While both CHIP and mCAs are risk factors for developing a subsequent hematologic malignancy, most individuals with CH do not develop a malignancy, motivating the need to identify high-risk CH subtypes^2,4–6,8,11–14^. Specifically, few studies have evaluated the potential synergistic role played by the co-occurrence or overlap of CH types, although preliminary observations suggest elevated risk of cancer and cancer-related phenotypes^2,3,15,16^. In the present study, we characterized the frequency of co-occurring CH in a large, population-based analysis comprising three studies: the UK Biobank (UKBB, *N* = 478,441) for our initial discovery analyses, the Trans-Omics for Precision Medicine Program (TOPMed, *N* = 67,390), and the NCI’s Prostate, Lung, Colorectal and Ovarian Cancer Screening Trial (PLCO, *N* = 259) as independent replication sets. We identified 210 CH co-occurrences enriched over what is expected by chance, characterized associations of 28 CHIP-mCA co-occurrences with cancer-related phenotypes, and nominated high-risk clones associated with hematologic cancer risk.

## Results

### Characterization of CHIP in cancer-free UKBB participants

**Fig. 1:**
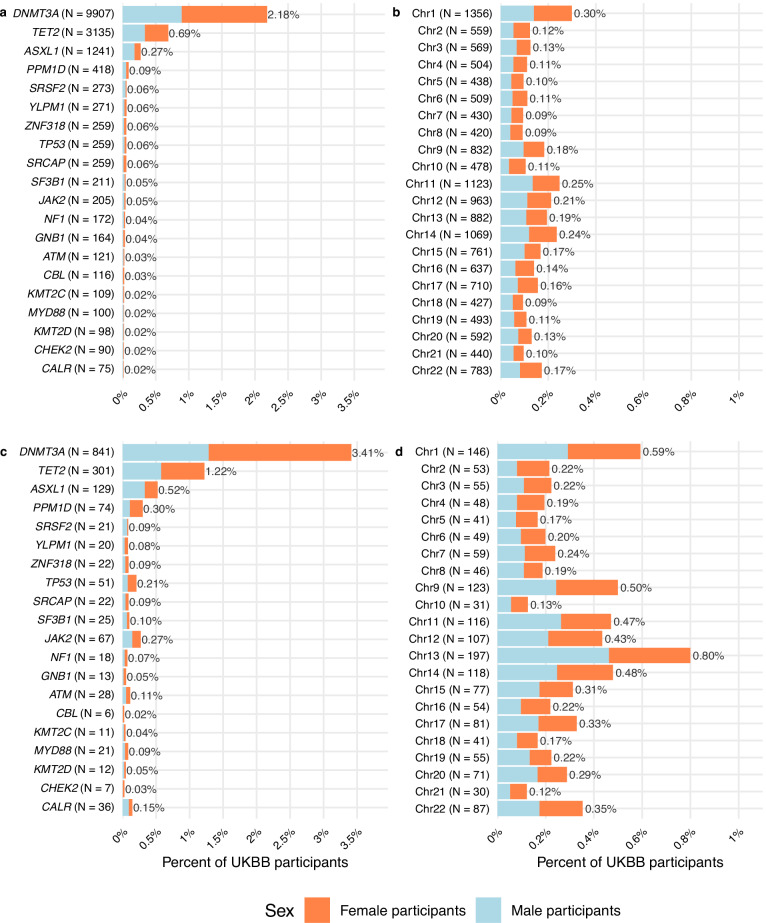
Frequencies of CHIP and autosomal mCA events among UK Biobank participants with and without prior cancer at baseline. **a** Percentage of participants without prior cancer with detectable clonal hematopoiesis of indeterminate potential (CHIP), stratified by sex (female: orange; male: blue). **b** Percentage of participants without prior cancer with autosomal mCAs. **c** Percentage of participants with prior cancer with detectable CHIP. **d** Percentage of participants with prior cancer with detectable autosomal mCAs. Percentages indicate the fraction of participants within each group (*N* = 453,807 without prior cancer; *N* = 24,634 with prior cancer).

A total of 21,737 CHIP mutations were detected in 20,354 UKBB participants with no prior cancer diagnosis (4.5%; Supplementary Data 1). CHIP was detected in 533 genes, with *DNMT3A, TET2*, and *ASXL1* variants present in 70.2% of cancer-free participants with detectable CHIP (Fig. 1a). All subsequent co-occurrence analyses were restricted to CHIP genes harbored by at least 30 UKBB participants to ensure adequate sample size, resulting in 43 genes for investigation. This criterion included 18,693 CHIP events detected in 17,865 UKBB participants. The number of detected mutations in individuals with CHIP ranged from 1 to 7.

### Characterization of mCAs in cancer-free UKBB participants

A total of 71,246 mCAs were detected in 67,081 UKBB participants with no prior cancer diagnosis (14.8%; Supplementary Data 1). The number of detectable mCAs per individual with mCAs ranged from 1 to 14. mLOY was the most frequent mCA detected in males (*N* = 41,432, 19.8% of males), and mLOX was most common in females (*N* = 14,839, 6.1% of females). A total of 14,975 autosomal mCAs were detected in 13,422 (3.0%) UKBB participants, most frequently on chromosomes 1, 11, and 14 (Fig. 1b).

### Characterization of co-occurring CH in cancer-free UKBB participants

**Fig. 2:**
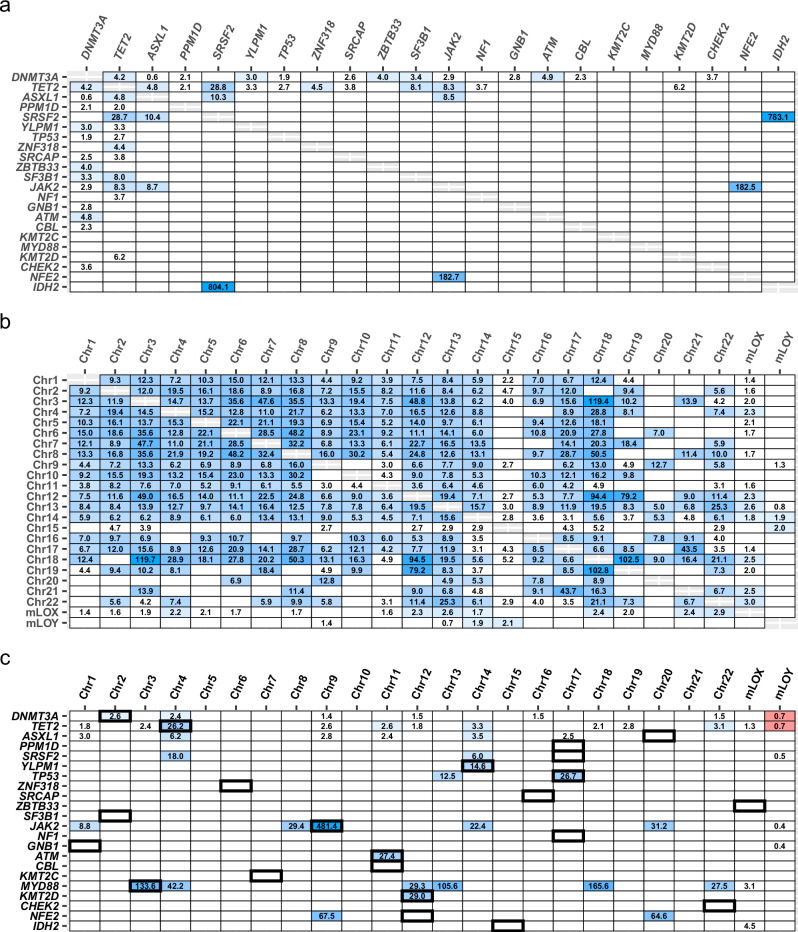
Enriched CH co-occurrences in UKBB participants without prior cancer at baseline. **a** Co-occurrence of mutations in common CHIP genes. **b** Co-occurrence of mCAs. **c** Co-occurrences of mutations in common CHIP genes and mCAs. Associations were tested using logistic regression adjusted for age, age^2^, sex, smoking status, and genetic ancestry; odds ratios (ORs) are shown for associations with *P* < 0.05 (two-sided tests). Colored cells indicate associations meet Bonferroni-corrected thresholds (**a**, *P* ≤ 2.7 × 10^−5^; **b**, *P* ≤ 8.7 × 10^−5^; **c**, *P* ≤ 4.8 × 10^−5^). Blue denotes OR > 1 with intensity proportional to effect size, and red OR < 1. Bold boxes indicate the chromosomal location of each CHIP gene. Full results are provided in Supplementary Data 2, 3 and 5.

We first examined co-occurrences within CH types (e.g., CHIP-CHIP or mCA-mCA co-occurrences) in the UKBB population. Of the 18,693 individuals with a detectable CHIP mutation in the 43 genes investigated in this study (*N* ≥ 30), we observed 957 (5.1%) individuals with CHIP in more than one gene. Most common co-occurrences were *DNMT3A-TET2* (*N* = 336), *TET2*-*SRSF2* (*N* = 65), and *TET2*-*ASXL1* (*N* = 60) (Supplementary Data 2). These co-occurrences were largely attributable to high frequencies of the individual CHIP mutations, resulting in joint acquisition of both events, which align with anticipated frequencies. To identify enrichment of specific co-occurrences, we tested if the frequency of the co-occurrences differed from expected frequencies derived from marginal frequencies, adjusting for age, age^2^, sex, smoking status, and genetic similarity to reference populations. We observed 14 CHIP-CHIP co-occurrences at higher frequencies than expected *(P* ≤ 2.7 × 10^−5^; Fig. 2a, Supplementary Data 2) with the strongest enrichment observed for *SRSF2*-*IDH2* (odds ratio (OR) = 804.1, 95% confidence interval (CI) = (430.8–1500.8), *P* = 5.2 × 10^−98^), followed by *JAK2-NFE2* (OR = 182.7, 95% CI = (76.6–435.8), *P* = 7.6 × 10^−32^) and *SRSF2-TET2* (OR = 28.8, 95% CI = (21.7–38.4), *P* = 5.4 × 10^−117^). Six genes (*DNMT3A*, *TET2*, *ASXL1*, *SRSF2*, *SF3B1*, *JAK2*) were found to have multiple significantly elevated CHIP-CHIP co-occurrences; for example, DNMT3A co-occurred at higher frequencies than expected with mutations in *TET2*, *YLMP1*, *ZBTB33*, *SF3B1*, and *ATM* (Fig. 2a). For *TET2* co-occurrences, many participants had more than one detectable *TET2* point mutation, ranging from 2 to 4, within the gene. Comparison of variant allele fractions (VAFs) among enriched CHIP-CHIP component events revealed no consistent patterns, suggesting the order of mutational acquisition did not favor more frequently observed CHIP mutations (e.g., *DNMT3A*, *TET2*, *ASXL1*). CHIP-CHIP co-occurrences were not observed significantly less frequently than expected, indicating no evidence for negative selection.

Of the 67,081 individuals with a detectable mCA, 3425 (5.1%) individuals harbored an mCA on more than one chromosome. Most common co-occurrences were chr14-mLOY and chr15-mLOY in males and chr22-mLOX and chr12-mLOX in females, largely attributable to the high frequencies of the component events (Supplementary Data 3). Out of the 286 unique mCA-mCA combinations, 168 (58.7%) had higher frequencies than expected (*P* ≤ 8.7 × 10^−5^) (Fig. 2b and Supplementary Data 3). Chr3-chr18 (OR = 119.7, 95% CI = (89.4–160.3), *P* = 1.9 × 10^−226^), chr18-chr19 (OR = 102.8, 95% CI = (74.0–142.8), *P* = 2.6 × 10^−168^), and chr12-chr18 (OR = 94.5, 95% CI = (73.0–122.3), *P* = 4.3 × 10^−261^) had the strongest effects (Supplementary Data 3) and often included whole chromosome amplifications of both chromosomes. Interestingly, other frequent mCA-mCA co-occurrences, such as chr12-chr19 (*N* = 74) and chr3-chr12 (*N* = 60), were also often comprised of whole chromosome gains. We then further refined our analysis to mCAs defined by chromosomal region and event type (e.g., chr9 p-arm CNLOH) and found that enriched co-occurrence patterns were largely consistent with increased magnitude of association. For example, co-occurrence of whole chromosome gains of chr3 and chr18 showed substantially greater enrichment (OR = 1014.6, 95% CI = (641.6–1604.5), *P* = 1.4 × 10^−192^) (Supplementary Fig. 1 and Supplementary Data 4). As with CHIP-CHIP co-occurrences, no mCA-mCA co-occurrences occurred less frequently than expected.

In an investigation of CHIP-mCA co-occurrences, we observed 3618 (0.8%) UKBB participants with both detectable CHIP and mCAs. Most frequent co-occurrences were *DNMT3A* with mLOY (*N* = 869) or mLOX (*N* = 444) as well as *TET2* with mLOY (*N* = 340), again attributable to the high frequencies of the component events (Supplementary Data 5). Interestingly, co-occurrences including mLOY occurred less frequently than expected (OR_*DNMT3A*_ = 0.7, 95% CI = (0.7–0.8), *P* = 9.5 × 10^−16^; OR_*TET2*_ = 0.7, 95% CI = (0.6–0.8), *P* = 4.5 × 10^−9^), suggesting possible exclusivity between these CH types (Supplementary Data 5). CHIP and autosomal mCAs were detected in 1165 (0.3%) UKBB study participants, representing approximately 2-fold more co-occurrences than expected (*N*_expected_ = 602, binomial *P* = 5.1 × 10^−92^). Twenty-eight (28) CHIP-autosomal mCA co-occurrences emerged from enrichment analyses *(P* ≤ 4.8 × 10^−5^) (Fig. 2c and Supplementary Data 5).

Co-occurrences were grouped into two categories for further analysis: overlapping and non-overlapping^3^. Overlapping CHIP-mCA events, where a CHIP mutation was detected within the bounds of a co-occurring mCA, were more frequent and observed in 314 participants (27.0% of UKBB participants with CHIP-autosomal mCA co-occurrences). This accounted for 8 enriched co-occurrences (*ATM-*chr11, *DNMT3A-*chr2, *JAK2-chr9, KMT2D*-chr12, *MYD88-*chr3, *TET2-*chr4, *TP53-*chr17, *YLPM1-*chr14) (Fig. 2c). Of these, *JAK2-*chr9 was detected most frequently (*N* = 98) and exhibited the greatest enrichment (OR = 481.4, 95% CI = (360.5–642.8), *P* < 2.2 × 10^−308^), followed by *MYD88-*chr3 (OR = 133.6, 95% CI = (78.9–226.0), *P* = 2.5 × 10^−74^) (Fig. 2c and Supplementary Data 5). We noted high consistency in the location and type of the CHIP mutation and mCA in participants with these two co-occurrences. For example, *JAK2*-chr9 co-occurrences were primarily *JAK2* V617F-chr9pCNLOH, and *MYD88*-chr3 co-occurrences were generally *MYD88* L252P-whole chromosome 3 amplifications. In other cases, we observed instances where the CHIP mutation within a gene varied, but the mCA was the same (e.g., *ATM* missense variants-chr11qCNLOH, *DNMT3A* missense variants-chr2pCNLOH). Similar to *TET2* CHIP-CHIP co-occurrences, individuals with *TET2*-chr4 co-occurrences often had more than one *TET2* point mutation. In analyses refined to include mCA chromosomal location and event types, we observed increases in the magnitude of association for many CHIP-mCA co-occurrences. For example, *JAK2*-chr9pCNLOH showed a stronger association (OR = 1741.1, 95% CI = (1261.3–2404.3), *p* < 2.2 × 10^−308^) (Supplementary Fig. 2 and Supplementary Data 6).

In UKBB participants with non-overlapping CHIP-mCA co-occurrences, we noted 20 enriched co-occurrences among 223 participants (19.3% of participants with both CHIP and autosomal mCAs), where the CHIP mutation was not detected within the bounds of a co-occurring mCA. The most notable co-occurrence observed was *MYD88-*chr18 (OR = 165.6, 95% CI = (96.5–284.3), *P* = 1.1 × 10^−76^), where *MYD88* L252P-whole chr18 gains predominated the specific events composing this co-occurrence (Fig. 2c and Supplementary Data 5). In refined analyses of mCA co-occurrence, *MYD88*-whole chr18 gains were further enriched (OR = 267.8, 95% CI = (141.1–508.3), *P* = 1.5 × 10^−65^); though CIs overlapped (Supplementary Fig. 2 and Supplementary Data 6). *MYD88*-chr13 also frequently co-occurred (OR = 105.6, 95% CI = (64.9–171.6), *P* = 7.7 × 10^−79^), primarily involving chr13q loss (Fig. 2c and Supplementary Data 5). 27 (93.1%) participants with non-overlapping enriched *JAK2-*mCA co-occurrences (*JAK2-*chr1, *JAK2*-chr8, *JAK2*-chr14, *JAK2*-chr20) had more than 1 mCA. Many participants (58.3%) with non-overlapping *ASXL1*-chr4qCNLOH also had overlapping *TET2*-chr4qCNLOH mutations, emphasizing the relationship between these two epigenetic regulators. No overlapping or co-occurring CHIP-autosomal mCA co-occurrences occurred less frequently than expected.

For enriched CHIP-mCA co-occurrences, we examined the clonal fractions of component CHIP and mCA events to provide insight into the clonal history of CH mutations. In general, estimated CHIP clonal fraction was higher than estimated mCA clonal fraction in the case of both overlapping (92.6%) and non-overlapping CH (86.7%) (Supplementary Fig. 3). The relationship was most pronounced in co-occurrences with *JAK2* CHIP mutations, suggesting that the acquisition of this CHIP mutation likely preceded the acquisition of the associated autosomal mCA; although these analyses were performed in the absence of longitudinal data and assume clones with higher observed fraction develop earlier.

### TOPMed replication of CH co-occurrences

A total of 3300 CHIP mutations were detected in 3019 (5.8%) TOPMed participants. Variant calling identified CHIP in 49 genes, as compared to 533 in the UKBB, reflecting the different CHIP calling and sequencing methods used for TOPMed (Methods). Similar to UKBB, CHIP mutations were most frequently detected in *DNMT3A, TET2*, and *ASXL1* (Supplementary Fig. 4a). In addition, a total of 9963 mCAs were detected in 9008 (13.4%) TOPMed participants. mCAs were frequently detected on the sex chromosomes (mLOY and mLOX) and chromosomes 11, 1, and 9 (Supplementary Fig. 4b).

Of the 3019 TOPMed participants with detectable CHIP, 245 (8.1%) had more than one detectable CHIP mutation. Most CHIP-CHIP co-occurrences were comparable to those observed in the UKBB, with 6 significantly enriched (*P* ≤ 8.7 × 10^−5^): *U2AF1-JAK2, SRSF2-TET2, JAK2-TET2, JAK2-ASXL1, ASXL1-TET2*, and *DNMT3A-TET2* (Supplementary Fig. 5a, Supplementary Fig. 6a, and Supplementary Data 7). *U2AF1-JAK2* had the greatest magnitude of enrichment (OR = 237.0, 95% CI = 68.8–816.8, *P* = 4.6 × 10^−18^) (Supplementary Fig. 5a and Supplementary Table 7). *TET2* mutations were again distributed across the gene and frequently observed as concurrent point mutations within individuals.

Investigating mCA-mCA co-occurrences in TOPMed, we noted that 754 individuals had more than one mCA, 414 of which had multiple autosomal mCAs. Enrichment testing identified 41 mCA-mCA co-occurrences that occurred more frequently than expected (*P* ≤ 8.7 × 10^−5^) (Supplementary Fig. 5b and Supplementary Data 8). This represents fewer enriched co-occurrences than observed in the UKBB, likely attributable to the difference in sample size of each cohort. Of these, 39 (95.1%) were also identified in the UKBB with a similar magnitude of enrichment (Supplementary Fig. 6b). The strongest enrichment was observed for co-occurrences that often involved whole chromosome gains, such as chr18-chr3 (OR = 70.5, 95% CI = 36.3–136.8, *P* = 3.0 × 10^−36^) and chr19-chr18 (OR = 67.6, 95% CI = 29.6–154.6, *P* = 1.6 × 10^−23^) (Supplementary Data 8). No mCA-mCA co-occurrences occurred less frequently than expected.

We examined CHIP-mCA co-occurrences, which were detected in 716 (1.4%) TOPMed participants. In agreement with UKBB results, we noted that *DNMT3A*-mLOY was observed less frequently than expected (OR = 0.6, 95% CI = (0.5–0.8), *P* = 2.7 × 10^−4^), though this association was no longer significant when corrected for multiple comparisons (Supplementary Fig. 5c and Supplementary Data 9). For CHIP and autosomal mCAs, 4 overlapping co-occurrences emerged from enrichment analyses (*P* ≤ 8.7 × 10^−5^): *DNMT3A*-chr2, *TET2*-chr4, *JAK2*-chr9, and *TP53*-chr17 (Supplementary Fig. 5c and Supplementary Data 9). Non-overlapping CH co-occurrences were also observed for *TET2*-chr14, *JAK2*-chr11, and *TET2*-chr9; the last two of which were not observed in UKBB. The greatest overall enrichment among CHIP-mCA co-occurrences in TOPMed was observed for *JAK2*-chr9 (OR = 193.6, 95% CI = (119.7–313.1), *P* = 3.4 × 10^−102^) (Supplementary Fig. 5c and Supplementary Data 9), replicating the striking enrichment observed in the UKBB (OR = 481.4); albeit with attenuated effect (Supplementary Fig. 6c). Most genomically overlapping co-occurrences consisted of partial arm CNLOH where the CHIP mutation was located (e.g., *DNMT3A-*2pCNLOH, *JAK2-*9pCNLOH, *TET2*−4qCNLOH). No CHIP-autosomal mCA co-occurrences were detected less frequently than expected in TOPMed.

### PLCO replication of CH co-occurrences

We performed exome sequencing on 259 PLCO participants with detectable mCAs. We detected CHIP mutations in 83 (32.0%) individuals. Observed CHIP-mCA co-occurrences included *ASXL1*-chr14, *DNMT3A-*chr2, *DNMT3A*-chr4, *MYD88*-chr3, *MYD88*-chr12, *MYD88*-chr13, *TET2-*chr11, and *TET2*-chr14, further replicating previously identified CH co-occurrences (Supplementary Data 10).

### Characterization of co-occurring CH in UKBB participants with prior cancer

We also characterized CH in 24,634 UKBB participants with a prior cancer diagnosis (Supplementary Data 11). A total of 1876 (7.6%) participants harbored 2115 CHIP events, which is 1.7 times the frequency of cancer-free individuals (Fig. 1c); this elevation remained significant in multivariable models adjusted for potential confounders (OR = 1.4, 95% CI = (1.3–1.5), *P* = 3.4 × 10^−40^). *CALR*, *JAK2, ATM, MYD88, PPM1D, TP53, KMT2D, NF1, ASXL1, TET2*, and *DNMT3A* CHIP mutations were more frequently observed in prior cancer cases (Supplementary Data 12). Autosomal mCAs were also detected at a higher frequency, with 1289 participants (5.2%) harboring 1685 events (OR = 1.5 compared to participants with no prior cancer) (Fig. 1d). mCAs on 20 autosomes were more frequent, with chr13 mCAs, composed primarily of losses, showing the greatest increase in frequency (OR = 3.5, 95% CI = 2.9–4.0, *P* = 8.9 × 10^−54^) (Supplementary Data 12).

Co-occurring CH was also more common in prior cancer cases (3.6%) with patterns similar to those without prior cancer; although sample size was reduced for identifying enriched co-occurrences. All previously identified co-occurring CHIP-CHIP mutations (*DNMT3A-TET2, TET2-SRSF2, TET2-JAK2*) were also observed in the prior cancer population (Supplementary Fig. 7a and Supplementary Data 13). Eighty-eight (30.8%) of 286 unique mCA-mCA co-occurrences reached statistical significance (*P* ≤ 8.7 × 10^−5^) (Supplementary Fig. 6b and Supplementary Data 14). Of the 28 CHIP and autosomal mCA co-occurrences previously described, 8 were enriched in prior cancer cases (Supplementary Fig. 7c and Supplementary Data 15). *TP53*-chr17 mCAs were more frequent in the prior cancer population (OR = 60.8, 95% CI = (27.2–135.8), *P* = 1.2 × 10^−23^) compared to those with no prior cancer (OR = 26.6, 95% CI = (20.8–33.0), *P* = 5.4 × 10^−28^); although the difference was not statistically significant (Supplementary Fig. 8). In contrast, *JAK2*-chr9 co-occurrences were detected less frequently in those with prior cancer (OR = 209.3, 95% CI = 121.2–361.5, *P* = 6.8 × 10^−82^, Supplementary Fig. 8). Two enriched CHIP-mCA co-occurrences emerged in the prior cancer population that were not previously identified (*SF3B1-*chr13 and *TP53*-chr14) (*P* ≤ 4.8 × 10^−5^) (Supplementary Fig. 7c and Supplementary Data 15).

### Risk factors for CH co-occurrences

**Fig. 3:**
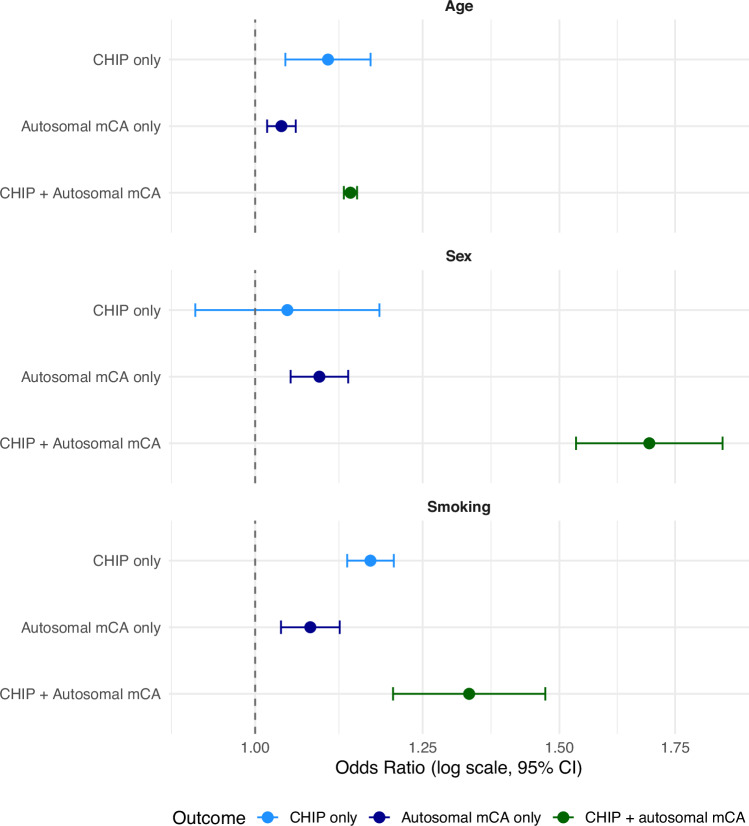
Risk factors associated with CHIP-autosomal mCA co-occurrence. Associations between participant characteristics (age, sex, and smoking status) and three CH categories: CHIP only (light blue), autosomal mCA only (dark blue), and CHIP and autosomal mCA co-occurrence (green). Points represent odds ratios (ORs) and error bars represent 95% confidence intervals (CIs) from multivariable logistic regression models adjusted for age, age^2^, sex, smoking status, and genetic similarity, where appropriate. Sex-related estimates use females as the reference group, and smoking estimates compare ever versus never smokers. Analyses were performed at the level of individual participants. Estimates are derived from meta-analyses of UKBB (*N* = 453,807) and TOPMed (*N* = 67,390); all tests are two-sided. Full results are provided in Supplementary Data 16.

To investigate whether participant characteristics associated with CHIP or autosomal mCAs (e.g., age, sex, smoking status) were associated with CHIP-autosomal mCA co-occurrence, we performed meta-analyses of UKBB and TOPMed data. Increasing age exhibited the most significant association (OR = 1.14 per 1 year increase in age, 95% CI = (1.13–1.15), *P* = 2.5 × 10^−174^), though the effect size was comparable to that observed for CHIP only (Fig. 3 and Supplementary Data 16). In contrast, ever smoking (OR = 1.3, 95% CI = (1.2–1.5), *P* = 3.6 × 10^−8^) and male sex (OR = 1.7, 95% CI = (1.5–1.9), *P* = 5.8 × 10^−26^) were more strongly associated with CHIP-autosomal mCA co-occurrence than either CH type alone (Fig. 3 and Supplementary Data 16). BMI was modestly inversely associated with co-occurrence (OR = 0.98, 95% CI = (0.97–1.00), *P* = 6.4 × 10^−3^) (Fig. 3 and Supplementary Data 16).

### The effect of co-occurring CH on telomere length

**Fig. 4:**
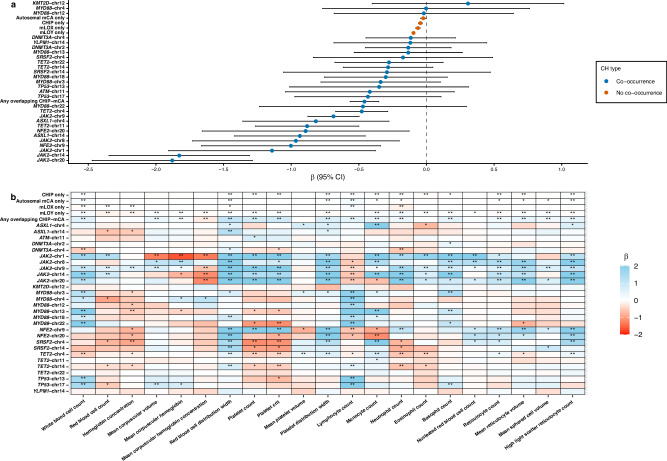
Associations between clonal hematopoiesis (CH) types, leukocyte telomere length, and hematopoietic-related phenotypes. Analyses were performed in UK Biobank participants without prior cancer (*N *= 453,807). Sample sizes for each CH category vary. **a** Association between CH types and leukocyte telomere length (LTL). Points show $\beta$ estimates, and error bars represent 95% confidence intervals (CIs) from multivariable linear regression models adjusted for age, age^2^, sex, smoking status, and genetic similarity. **b** Associations between CH types and 22 hematopoietic traits. Colors represent standardized (mean = 0, s.d. = 1) $\beta$ estimates from multivariable linear regression models adjusted as in (**a**). $\beta$ estimates are capped at $\left|\beta \right|=2$ for display. Asterisks indicate statistical significance (**P ≤ 3.33 × 10^−03^, multiple-testing threshold; *P > 3.33 × 10^−03^ and *P* ≤ 0.05; two-sided tests). Full results are provided in Supplementary Data 17.

We evaluated the combined effects of CH co-occurrences relative to their individual component events and to individuals without detectable CH on measured leukocyte telomere length (LTL), an indicator of cellular replication, in UKBB participants without prior cancer. We observed evidence for telomere degradation in individuals with overlapping CHIP-mCA events ($\beta$ = −0.46, 95% CI = (−0.56 to −0.35), *P* = 2.0 × 10^−17^), with 11 enriched CHIP-mCA co-occurrences associated with shorter telomeres (Fig. 4a and Supplementary Data 17). Individuals with *JAK2*-chr20 harbored the shortest telomeres ($\beta$= −1.9, 95% CI = (−2.5 to −1.3), *P* = 5.1 × 10^−10^) (Fig. 4a and Supplementary Data 17).

We next compared the effect of co-occurrences to their component events (e.g., *JAK2*-chr9 vs. *JAK2* CHIP only or chr9 mCA only) to identify potential compounded effects of CH co-occurrences. For example, telomere shortening was observed in participants with *JAK2* only ($\beta$ = −0.4, 95% CI = −0.6 to −0.2, *P* = 7.5 × 10^−4^) and chr9 mCAs only ($\beta$ = −0.1, 95% CI = (−0.2 to −0.02), *P* = 1.4 × 10^−2^) (Supplementary Data 17); however, those with overlapping *JAK2*-chr9 co-occurrences had substantially shorter telomeres ($\beta$ = −0.8, 95% CI = (−0.9 to −0.5), *P* = 1.1 × 10^−12^) (Supplementary Table 17).

### Hematopoietic-related phenotypes in individuals with CHIP and mCAs

Based on established reference ranges, we determined the percentage of UKBB participants without a prior cancer who had blood counts outside normal hematologic ranges to identify potential biological impacts of CHIP-mCA co-occurrences relative to component events. Among individuals without CH, 21% had abnormal white blood cell, red blood cell, or platelet counts. Similar elevated proportions were observed in those with CHIP only (23%) or mCAs only (23%) (Supplementary Fig. 8). In contrast, 52% of participants with overlapping co-occurrences had cell counts outside these ranges, including 66% of individuals with *JAK2*-chr9 (Supplementary Fig. 9).

For myeloid lineage traits, the greatest effects were observed for CH co-occurrences including *ASXL1*, *JAK2, NFE2, SRSF2*, or *TET2. JAK2* co-occurrences (*JAK2*-chr1, *JAK2*-chr9, *JAK2*-chr14, *JAK2-*chr20) had significantly elevated platelet counts, with *JAK2*-chr9 participants having the most elevation ($\beta$ = 2.7, 95% CI = (2.5–2.9), *P* = 1.0 × 10^−159^) (Fig. 4b and Supplementary Data 17). Participants with *JAK2* co-occurrences also had notable increases in monocyte, neutrophil, eosinophil, and basophil counts. In contrast, participants with *TET2* co-occurrences had decreased platelet, neutrophil, and eosinophil count (Fig. 4b and Supplementary Data 17). When comparing the effect of a co-occurrence compared to their component CHIP and mCA events, co-occurrences impacted myeloid lineage traits to a greater degree (Supplementary Data 17). Furthermore, participants harboring co-occurrences with high mCA cellular fraction (≥0.1) generally had greater alterations in myeloid lineage traits. For example, participants with high cellular fraction *JAK2-*chr14 co-occurrences had evidence for higher platelet counts than those with low cellular fraction *JAK2*-chr14 co-occurrences ($\beta$_high_cf_ = 1.5, 95% CI = (0.8–2.3), *P* = 8.5 × 10^−5^;$\,\beta$_low_cf_ = 1.1, 95% CI = (0.4–1.8), *P* = 2.0 × 10^−3^) (Supplementary Data 17).

For lymphoid lineage traits, we noted 13 CH co-occurrences with significant effects (*P* ≤ 3.33 × 10^−3^) (Fig. 4b and Supplementary Data 17). *MYD88* co-occurrences were associated with increased lymphocyte counts with standardized effect sizes ranging from 1.2 to 6.0 (Supplementary Data 17). In comparing these effect sizes to component events, *MYD88* CHIP only and chr13 mCAs had associations with increased lymphocytes ($\beta$= 1.8, 95% CI = (1.6–1.9), *P* = 9.8 × 10^−85^; *β* = 1.4, 95% CI = (1.4–1.5), *P* < 2.2 × 10^−308^, respectively), but the effect was profoundly stronger for *MYD88*-chr13 co-occurrences (*β* = 8.4, 95% CI = (8.1–8.6), *P* < 2.2 × 10^−308^) (Supplementary Data 17). Furthermore, participants with high cellular fraction *MYD88*-chr13 had higher elevations in lymphocyte counts compared to those with low cellular fraction *MYD88* ($\beta$_high_cf_ = 10.9, 95% CI = (10.6–11.2), *P* < 2.2 × 10^−308^;$\beta$_low_cf_ = 1.6, 95% CI = (1.1–2.1), *P* = 1.1 × 10^−10^) (Supplementary Data 17). Interestingly, participants with 3 of the 5 *JAK2* co-occurrences also had significant associations with lymphocyte count; however, in this case, lymphocyte count decreased (e.g., *JAK2*-chr9:$\beta$= −0.39, 95% CI = (−0.51 to 0.27), *P* = 9.5 × 10^−11^) (Supplementary Data 15).

### Myeloid malignancy risk

During follow-up, 1135 (0.3%) UKBB participants without prior cancer developed an incident myeloid malignancy. Of these, 531 (47%) individuals had detectable CH at baseline. As anticipated, participants with CHIP only had the greatest risk of myeloid malignancy compared to those with autosomal mCAs only (hazard ratio (HR)_CHIP_only_ = 5.5, 95% CI = (4.8–6.4), *P* = 6.6 × 10^−113^; HR_mCA_only_ = 3.0, 95% CI = (2.3–3.8), *p* = 2.2 × 10^−18^) (Supplementary Data 18). For general CH co-occurrences, risk was notably elevated in those with CHIP-CHIP co-occurrence compared to those with mCA-mCA co-occurrence (HR_CHIP-CHIP_ = 36.4, 95% CI = (29.3–45.2), *P* = 6.4 × 10^−230^). Overlapping CH conferred the greatest risk (HR = 80.6, 95% CI = (61.7–105.4), *P* = 9.78 × 10^−226^), exceeding that of those with CHIP and any autosomal mCA (HR = 38.0, 95% CI = (31.8–47.3), *P* = 6.3 × 10^−288^) (Supplementary Data 18).

**Fig. 5:**
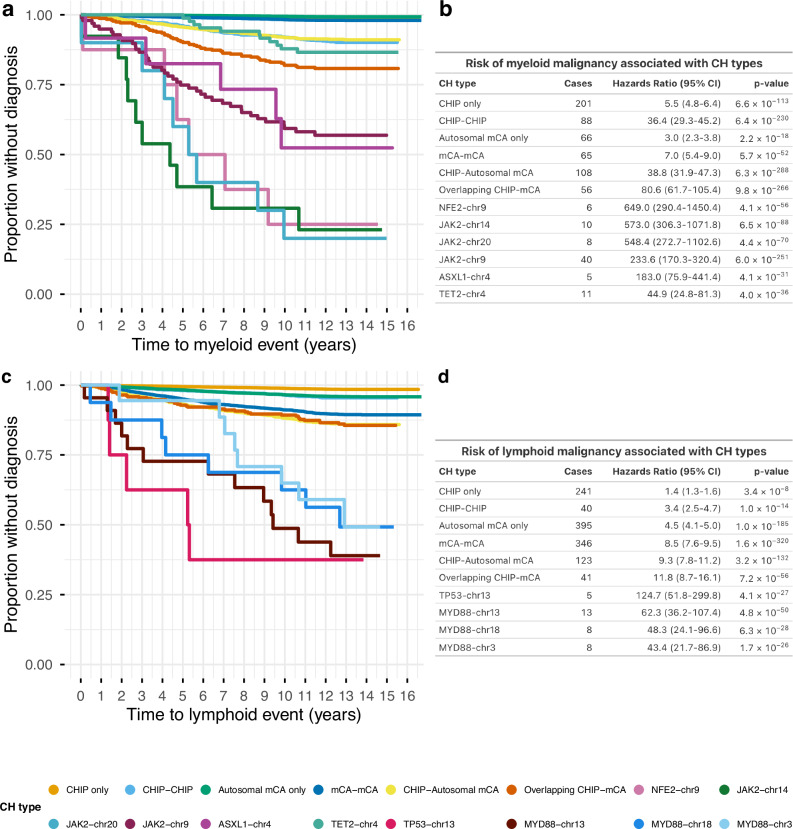
Risk of myeloid and lymphoid malignancy associated with CH types. CHIP-mCA co-occurrences shown had ≥5 cases, hazards ratio (HR) ≥ 40, and *P* ≤ 7.9 × 10^−4^. **a** Kaplan–Meier curves of time to myeloid malignancy by CH type. **b** HRs and 95% confidence intervals (CIs) for myeloid malignancy from Cox proportional hazards models adjusted for age, age^2^, sex, smoking status, and genetic similarity; two-sided Wald test *P* values are shown. **c** Kaplan–Meier curves of time to lymphoid malignancy by CH type. **d** HRs and 95% CIs for lymphoid malignancy from Cox proportional hazards models adjusted as in (**b**); two-sided Wald test *P* values are shown. Full results are provided in Supplementary Data 18 and 19.

Of the 28 enriched CHIP-mCA co-occurrences, 6 were shown to have elevated risk of myeloid malignancies (*P* ≤ 8.8 × 10^−4^): *ASXL1*-chr4, *JAK2*-chr9, *JAK2*-chr14, *JAK2*-chr20, *NFE2*-chr9, and *TET2*-chr4. Individuals with *NFE2*-chr9 co-occurrences (*N*_cases_ = 6) had the most extreme risk (HR = 649.0, 95% CI = (290.4–1450.4), *P* = 4.09 × 10^−56^), with a median time-to-event (TTE) of 5 years (Fig. 5b). *JAK2* co-occurrences had the next strongest risk, with *JAK2*-chr14 co-occurrences (N = 10) with the shortest median TTE of 2.9 years (HR = 573.0, 95% CI = (306.3–1071.8), *P* = 6.5 × 10^−88^) (Fig. 5a and Supplementary Data 18). Overlapping *JAK2*-chr9 (*N*_cases_ = 40) had a median TTE of 4.2 years (HR = 233.6, 95% CI = (170.3–320.4), *P* = 6.0 × 10^−251^) (Fig. 5b and Supplementary Data 18).

When comparing the risk of co-occurrences to component events, it was noted that risk is further potentiated by co-occurring CH. For example, participants with *JAK2* V617F only (*N* = 75) were at elevated risk (HR = 225.0, 95% CI = (154.75–327.14), *P* = 5.8 × 10^−177^); however, individuals with *JAK2-*mCA co-occurrences had higher estimates of risk (Supplementary Data 18). In comparison, participants with only chr9 or chr14 mCAs had substantially lower HRs of 27.2 and 5.0, respectively (Supplementary Data 18).

We additionally evaluated whether myeloid malignancy risk differed by cellular fraction in individuals with *JAK2*-chr9, the only co-occurrence with sufficient sample size to support this analysis. Although the hazard ratio was higher for individuals with high cellular fraction *JAK2*-chr9 events (*N*_cases_ = 73; HR = 230.1, 95% CI = (160.0–331.0), *P* = 5.8 × 10^−189^), the confidence intervals directly overlapped with those of the low cellular fraction group (*N*_cases_ = 25; HR = 218.1, 95% CI = (117.1–406.4), *P* = 1.6 × 10^−64^), suggesting similar risk estimates (Supplementary Data 18).

Lastly, we conducted sensitivity analyses excluding participants with white blood cell or platelet counts that deviated substantially from normal reference ranges to account for potential undiagnosed disease at baseline (Methods). Co-occurrences previously nominated as high risk remained significantly associated with similar estimates of risk (Supplementary Data 18).

### Lymphoid malignancy risk

A total of 4319 (1.0%) UKBB participants who were cancer-free at baseline developed an incident diagnosis of a lymphoid malignancy; 1614 (37.3%) of whom had detectable CH at baseline. As anticipated, individuals with autosomal mCAs only had a greater risk of lymphoid malignancies compared to those with CHIP only (HR_mCA_only_ = 4.5, 95% CI = (4.1–5.0), *P* = 1.0 × 10^−185^; HR_CHIP_only_ = 1.4, 95% CI = (1.3–1.6), *P* = 3.4 × 10^−8^) (Supplementary Data 19). Of general CH co-occurrences, risk was greater for those with mCA-mCA co-occurrence compared to those with CHIP-CHIP co-occurrence (HR_mCA-mCA_ = 8.5 vs. HR_CHIP-CHIP_ = 3.4) (Fig. 5d). Risk of lymphoid malignancies was again further elevated in individuals with overlapping CH, compared to those with CHIP and any autosomal mCA (HR = 11.8, 95% CI = (8.7–16.1), *P* = 7.2 × 10^−56^) (Fig. 5d).

Of the 28 enriched CH co-occurrences, 4 were shown to have elevated risk of lymphoid malignancies: *MYD88*-chr3, *MYD88*-chr13, *MYD88*-chr18, and *TP53-*chr13. Participants with *TP53*-chr13 (*N*_cases_ = 5) had the shortest median TTE of 2.2 years, as well as the highest risk (HR = 124.7, 95% CI = (51.9–299.8), *P* = 4.1 × 10^−27^) (Fig. 5c, d). *MYD88*-chr13, *MYD88*-chr18, and *MYD88*-chr3 had the next greatest risk associations (HRs = 43.4-62.3; *N*_cases_ = 13, 8, 8, respectively) (Fig. 5d). Of these, *MYD88*-chr18 participants had the shortest median TTE of 5.2 years (Fig. 5c).

In comparing the risk of these co-occurrences to their component events, we noted that participants with *TP53* only had negligible risk of lymphoid malignancy (HR = 1.6, 95% CI = (0.6–4.3), *P* = 0.3) and participants with only chr13 mCAs had moderate levels of risk (HR = 16.5, 95% CI = (13.1–20.7), *P* = 6.2 × 10^−127^) compared to those with *TP53*-chr13 (Supplementary Data 19). As such, it was unanticipated that these co-occurrences had such amplified risk (HR = 124.7). We were unable to evaluate lymphoid malignancy risk by mCA cellular fraction due to insufficient sample size. Similar to myeloid malignancy risk estimates, those co-occurrences associated with increased risk of lymphoid malignancy remained associated with high risk in sensitivity analyses that removed individuals with elevated white blood cell and platelet counts (Methods, Supplementary Data 19).

## Discussion

Understanding the progression of detectable CH clones to malignant states is crucial in light of the prevalence of CH in aging adult populations. Most prior studies of CH have examined either CHIP or mCAs, limiting the ability to assess the combined effects of co-occurring or overlapping CH^4,5,17–19^. Our integrative analysis of detectable CHIP and mCAs in UKBB, TOPMed, and PLCO identified co-occurring CH in approximately 1.6% of adults in the general population. We characterized previously unreported co-occurrences and observed strong evidence for enrichment of co-occurrence, with some clones detected orders of magnitude more frequently than anticipated. Our findings detail the persistence of highly selected co-occurring clones. We identified alterations in telomere length and hematological parameters, as well as pronounced elevations in risk of myeloid and lymphoid malignancies for specific CH co-occurrences.

Our results build on prior observations of CH co-occurrence^2,3,15,16,20^. First, our findings demonstrate that the co-occurrence of multiple CH types within a single clone is not only tolerated but may also confer a selective advantage, demonstrated by the substantially higher frequencies of certain CH co-occurrences relative to the marginal frequencies of individual events. Overlapping CH could be part of a “two-hit” model at the genomic locus in which normal copies of a gene are lost^21^. Clonal fraction data indicate that CHIP events are present at a higher cell fraction and likely occur first at the locus. This first coding driver mutation could confer an initial selective advantage to the clone. Subsequently acquired mCAs at the locus can delete the normal copy of the gene or amplify the mutated copy, resulting in a clone with amplified selective potential. Genetic mechanisms selective for non-overlapping CH events are less clear but potentially impact multiple interrelated genes within a pathway to confer a selective advantage. Some CH co-occurrences, however, are observed less frequently than expected, as is the case for *DNMT3A*-mLOY. The reduction in frequency of such co-occurrences suggests potential clonal competition or that the presence of both events is not tolerated within a single clone.

Second, CH co-occurrence has higher rates of cellular expansion than either component CHIP or mCA events. This is observed not only in higher cell fractions of co-occurring clones but also through greater reductions in telomere length, an indicator of cellular replication. This finding indicates cells with co-occurring CH can support the necessary cellular replication to expand and maintain this clonal expansion. Such a predilection for clonal expansion could lead to concerning growth trajectories as well as promote increased genomic instability that predisposes to the acquisition of additional mutations that further drive growth and expansion.

Third, the clonal expansion of co-occurring CH leads to marked phenotypic manifestations. We noted key disruptions in the balance of the hematopoietic compartment in individuals with co-occurring CH. Many imbalances were lineage-specific, such that some co-occurrences led to more pronounced alterations in the myeloid compartment, whereas others were more associated with alterations in lymphoid lineage traits. Several investigated co-occurrences conferred greater hematopoietic alterations than the cumulative effects of their component events, further confirming the importance of assessing CHIP and mCAs simultaneously.

Finally, our analysis of a large prospective cohort enabled the compilation of a list of CH co-occurrences that are associated with a high risk of progression to hematologic malignancies with hazard ratios exceeding 40. The six CH co-occurrences that confer such risk of myeloid malignancy progression include *ASXL1*-chr4, *JAK2*-chr9, *JAK2*-chr14, *JAK2*-chr20, *NFE2*-chr9^22,23^, and *TET2*-chr4. The CH co-occurrences that confer such risk of lymphoid malignancy progression include *MYD88*-chr3, *MYD88*-chr13, *MYD88*-chr18, and *TP53-*chr13. Surveillance for the presence and expansion of these clones could enable earlier detection and intervention in patients at risk of hematologic malignancies, especially in light of the short time to events observed for some co-occurrences.

Although the co-occurrence of CHIP and mCAs is suggestive of shared clonal origin, particularly for events detected at overlapping genomic loci, our analyses were based on bulk sequencing and genotyping data and therefore cannot definitively resolve clonality. In instances where CHIP mutations and mCAs are detected at low cellular fractions, it remains possible that these alterations arose in independent clones within the same individual. Nevertheless, the magnitude of enrichment observed for many of the identified CH co-occurrences is more consistent with a shared clonal origin than with independent clonal events occurring by chance. Accordingly, our findings should be interpreted as population-level evidence of non-random co-occurrence that is consistent with, but does not conclusively establish, shared clonal architecture.

A few limitations of this study warrant consideration. Differences in CH detection methods across UKBB, TOPMed, and PLCO could introduce variability in the sensitivity for low-frequency events and alterations in specific genomic regions not captured well in the library preparation. Despite this being the largest study of its kind, limited sample sizes for many co-occurrences may reduce our power to identify associations and affect the precision of risk measurements. The cross-sectional assessment of CH limits our ability to examine the temporality in the acquisition of CH event types and interpret patterns of clonal evolution. In comparing VAF and cellular fraction, we did not account for local ploidy, which may further confound interpretations of mutational timing. However, the prospective nature of the UK Biobank was a key strength for identifying potential associations with future malignancy risk. Although the inclusion of TOPMed improved representation of individuals similar to non-European ancestry reference groups, the generalizability of our findings to these populations remains limited. Lastly, we acknowledge the possibility of undiagnosed hematologic malignancies at baseline, which could bias hazard ratios due to the inclusion of individuals with pre-existing but clinically unrecognized disease. Sensitivity analyses were performed that removed individuals with elevated white blood cell and platelet counts to address this potential bias.

Our findings highlight the importance of remaining vigilant to the complexities of multiple concurrent CH events, the forces governing selection and clonal expansion of these clones, and the multitude of potential phenotypic consequences. Future population-level, multi-omic, and clinical research is crucial for further disentangling the clinical relevance of co-occurring CH clones.

## Methods

All studies included in this work were conducted in accordance with relevant ethical regulations and were approved by the appropriate institutional review boards (IRBs) or ethics committees. All participants provided written informed consent. Study-specific details regarding ethical approvals and consent procedures are provided in the corresponding cohort descriptions and references.

### UK Biobank

#### Ethics statement

The research conducted in this study complies with all relevant ethical regulations. UK Biobank (UKBB) has approval from the Northwest Multi-centre Research Ethics Committee (MREC) as a Research Tissue Bank (RTB) approval. All participants provided informed consent to participate in the UK Biobank.

#### Study participants

The UK Biobank is a prospective study with more than half a million participants between 37 and 73 years of age. Participants were recruited between 2006 and 2010 in 22 assessment centers throughout the UK. Baseline assessment visits were comprised of electronic consent, a touch-screen questionnaire, a computer-assisted interview, physical and functional measurements, and the collection of specimens, including blood, urine, and saliva. A more in-depth description of the UK Biobank study design and cohort is provided by Sudlow et al., as well as at https://www.ukbiobank.ac.uk^24^. Within the present study, analyses were performed on 453,807 UKBB subjects, after removing those who had withdrawn from the study, failed genotyping QC, or exhibited sex discordance. In addition, those with reported cancer (ICD-10 C codes) within inpatient and cancer registry records prior to study enrollment were removed from primary analyses but were later evaluated in analyses restricted to cancer survivors. Genetic similarity to 1000 Genomes reference populations (CEU, YRI, ASN) was estimated in SNPweights to adjust for confounding effects by ancestry^25^.

#### Detection of CH

For CHIP, somatic mutations were called within whole-exome sequencing (WES) data using the Artifact filtering Clonal Hematopoiesis (ArCH) variant calling pipeline for detecting SNVs and short insertions/deletions (INDELs)^26^. Mutect2 (v4.2.1.0)^27^, VarDict (v1.6.0) were used for variant calling, following base quality score recalibration with GATK’s BQSRPipelineSpark (v4.2.0.0)^27–29^. Variants supported by both variant callers, with a variant allele fraction (VAF) > 2% and at least 2 supporting reads from forward and reverse strands, were retained. To remove potential false positive calls due to sequencing error and artifact, we performed extensive filtering. Fisher’s exact tests were performed to test that mutant read depths were significantly higher relative to a panel of normal samples (PON) (*N* = 50) under the age of 21 without any prior known CH hotspot mutations. *P* values were required to reach stringent Bonferroni-correction thresholds based on the number of bases in the exome capture design to be considered significant. A recurrent filter was applied such that variants appearing in >10% of the samples were flagged as potential sequencing errors. Pre-compiled lists of CH driver genes were used to rescue potential variants flagged by the recurrent filter. Additionally, known germline mutations as annotated in the gnomAD database with allele frequency greater than 0.0005 were filtered out as potential germline variants. Further filters removed low VAF Mutect2 calls, synonymous mutations, variants with high strand bias, variants not reaching minimum alternative allele counts, and homopolymers regions. Only variants passing all filters and classified as putative drivers were retained in the final analytic set of CHIP variants. Variants were classified as putative drivers based on knowledge from COSMIC, oncoKB, and ClinVar databases, as well as utilizing a list of previously identified putative driver mutations and rules in Bolton et al. and Bick et al.^30,31^. Finally, all CHIP analyses were restricted to genes mutated in at least 30 participants (*N* = 43), as power was limited to assess genes less frequently impacted by CHIP. For clonal fraction comparison, CHIP cellular fraction was calculated by 2 × VAF, with a ceiling of 1.0; although this assumes no chromosomal losses or gains spanning the variant.

Previous studies have described the detection of mCAs in the UKBB^8,17^. DNA was extracted from baseline peripheral blood samples, followed by genotyping on the UK BiLEVE or UK Biobank Axiom array. Genotype intensities were transformed to log2R ratio (LRR) and B-allele frequency (BAF) values to estimate relative allelic intensities and allelic imbalances. Long-range phase information was utilized for assigning the phase for BAF deviations. MoChA, an mCA detection algorithm which employs a three-state hidden Markov model to analyze LRR and BAF data, in conjunction with genetic phase, was utilized to detect mCAs (https://github.com/freeseek/mochawdl), assign copy number state, and estimate cellular fraction.

For all subsequent analyses, the presence of both CHIP and mCAs was treated as binary. Individuals were classified as either having (1) or not having (0) each event type, regardless of the number of co-occurrences. For CHIP, this was at the gene level; for mCAs, this was at the chromosome level. Additionally, all *P* values from subsequent statistical analyses were derived from two-sided tests.

#### Characterization of co-occurring CH

Upon detection of both mCAs and CHIP within the population, data were merged to determine co-occurrence and overlap. Logistic regression models were used to examine if co-occurrences were more frequent than expected based on individual characteristics. Models were fit using the glm() function in R (v4.3.1) and were adjusted for age, age^2^, sex, smoking status (categorized as never, former, or current), and genetic similarity. ORs were estimated by exponentiating the regression coefficient from these models. Corresponding 95% CIs were calculated by exponentiating the coefficient ± 1.96 ×  its standard error (SE). Co-occurring CH was categorized in three groups: CHIP-CHIP (co-occurrence of CHIP mutations in two different genes), mCA-mCA (co-occurrence of mCAs on two different chromosomes), and CHIP-mCA (co-occurrence of a specific CHIP mutation and an mCA). For analyses of CHIP-CHIP and mCA-mCA co-occurrence, regression models were constructed such that CHIP mutations in each gene and mCAs on each chromosome were tested as both a predictor and outcome variable. For CHIP-mCA analyses, CHIP was tested as the predictor of the mCA outcome variable. Given that our analyses were based on bulk sequencing and genotyping data, co-occurrence was defined as the presence of multiple CH types within the same individual. Overlapping CH was defined as a CHIP mutation falling within the bounds of a co-occurring mCA.

To address multiple comparisons, a Bonferroni threshold of significance for each co-occurring CH group (CHIP × CHIP, mCA × mCA, CHIP × mCA) was determined. This was calculated by 0.05/(number of groups in CH type 1 × number of groups in CH type 2). For example, within the co-occurring CHIP and mCA group, this was calculated by 0.05/(24 × 43). Any enriched CHIP-mCA co-occurrences with *P* ≤ 4.8 × 10^−5^ that were detected in at least 5 participants were selected for inclusion in subsequent phenotypic association analyses.

For refined mCA co-occurrence analyses, mCAs were additionally grouped by chromosome region (p, q, both, or whole chromosome) and event type (gain, loss, CNLOH). Logistic regression models were constructed analogously to the chromosome-level analyses, testing mCAs as both predictors and outcomes and adjusting for age, age^2^, sex, smoking status (never, former, current), and genetic similarity. Bonferroni-adjusted significance thresholds were determined based on the number of arm-level comparisons in which ≥5 participants exhibited co-occurrence. For mCA-mCA co-occurrences, this threshold was 0.05/102 = 4.9 × 10^−4^; for CHIP-mCA co-occurrences, this threshold was 0.05/66 = 7.6 × 10^−4^.

### Replication in TOPMed

#### Study participants

A total of 67,390 participants from 19 studies within the National Heart, Lung, and Blood Institute (NHLBI) Trans-Omics for Precision Medicine (TOPMed) program were included in our analyses: Genetics of Cardiometabolic Health in the Amish (Amish; *N* = 1109)^32^, Atherosclerosis Risk in Communities Study (ARIC, *N* = 3870)^33^, Barbados Genetics Asthma Study (BAGS, *N* = 980), Mount Sinai BioMe Biobank (BioMe, *N* = 9392)^34^, Coronary Artery Risk Development in Young Adults (CARDIA, *N* = 3293)^35^, Cleveland Family Study (CFS, *N* = 1281), Cardiovascular Health Study (CHS, *N* = 3517)^36^, Genetic Epidemiology of COPD Study (COPDGene, *N* = 10,050)^37^, Framingham Heart Study (*N* = 4007)^38^, Genetic Studies of Atherosclerosis Risk (GeneSTAR, *N* = 1733)^39^, Genetic Epidemiology Network of Arteriopathy (GENOA, *N* = 1157), Genetics of Lipid Lowering Drugs and Diet Network (GOLDN, *N* = 942), Hispanic Community Health Study – Study of Latinos (HCHS/SOL, *N* = 3857)^40^, Hypertension Genetic Epidemiology Network (HyperGEN, *N* = 1865), Jackson Heart Study (JHS, *N* = 3317)^41^, Multi-Ethnic Study of Atherosclerosis (MESA, *N* = 5222)^42^, The Vanderbilt Atrial Fibrillation Registry (VU_AF, *N* = 1085), Women’s Genome Health Study (WGHS, *N* = 108), and Women’s Health Initiative (WHI, *N* = 10,695)^43^. Genetic similarity proportions for the percentage of African, Asian, and European ancestry were inferred for each participant using GrafPop^44^. The cancer status of these participants was not available for all contributing studies. All studies received IRB approval, and informed consent was obtained from all participants.

#### Detection of CH

Whole genome sequencing was generated as part of the NHLBI TOPMed program^45^. Detection of CHIP and mCAs in TOPMed has been reported in prior studies^16,31^. A total of 25,051 males were interrogated for mLOY in TOPMed. mLOX was called as ChrX events, defined as losses by MoChA, that spanned at least 100 Mb in length of ChrX.

#### Characterization of co-occurring CH

We replicated the co-occurrence analysis in TOPMed using the same methodology as described for UKBB participants. CHIP and mCA data were merged, and logistic regression models, adjusted for age, age^2^, sex, smoking status (never or ever), genetic similarity, and TOPMed study, were fit using the glm function in R (v4.4.2). CHIP-CHIP, mCA-mCA, and CHIP-mCA co-occurrences were evaluated. For those including CHIP, 24 specific CHIP genes were chosen for inclusion, listed in Supplementary Data 20. To address multiple testing, a Bonferroni correction was again applied using a uniform threshold across co-occurrence types. This was calculated as 0.05/ (24 × 24) = 8.7 × 10^−5^.

### PLCO replication

#### Study participants

The Prostate, Lung, Colorectal and Ovarian Cancer Screening Trial (PLCO) was a controlled, prospective study conducted across multiple sites in the United States^46^. Between 1993 and 2001, 154,807 individuals between the ages of 55 and 74 were enrolled at 10 screening centers. Participants provided biospecimens and completed detailed surveys on demographic information, family and medical history, and lifestyle and dietary risk factors. For those in the screening arm, blood samples were collected at baseline and during annual follow-up visits for up to 5 years. Written informed consent was provided by all participants, and the study received approval from all relevant IRBs.

#### DNA preparation

Genomic DNA (200 ng per sample) was purified using Agencourt AMPure XP Reagent (Beckman Coulter Inc, Brea, CA, USA) following the manufacturer’s instructions. Adapter-ligated libraries were then prepared using the KAPA HyperPlus Kit (KAPA Biosystems, Wilmington, MA) in conjunction with Bioo Scientific NEXTflex™ DNA Barcoded Adapters (Bioo Scientific, Austin, TX, USA), according to the KAPA-provided protocol.

#### Pre-hybridization LM-PCR

Prior to hybridization, genomic DNA sample libraries were amplified via ligation-mediated PCR (LM-PCR). Each reaction included 20 μL of library DNA, 25 μL of 2x KAPA HiFi HotStart ReadyMix, and 5 μL of 10x Library Amplification Primer Mix, containing primers with the sequences: 5′-AATGATACGGCGACCACCGA-3′ and 5′-CAAGCAGAAGACGGCATACGA-3′. PCR conditions consisted of an initial denaturation at 98 °C for 45 s, followed by 5 cycles of 98 °C for 15 s, 60 °C for 30 s, and 72 °C for 30 s, with a final extension at 72 °C for 1 min. Reactions were maintained at 4 °C until further processing. Amplified sample libraries were cleaned using the Agencourt AMPure XP Reagent (Beckman Coulter Inc, Brea, CA, USA), according to the KAPA-provided protocol, and quantified using Quant-iT™ PicoGreen dsDNA Reagent (Life Technologies, Carlsbad, CA, USA).

#### Liquid phase sequence capture

Amplified sample libraries with unique barcodes were pooled in equal amounts to form 1.1 μg input for multiplexed sequence capture. Exome sequence capture was performed using NimbleGen’s SeqCap EZ Human Exome Library, Exome+UTR with 96 Mb targeted (Roche NimbleGen, Inc., Madison, WI, USA). Prior to hybidriziation, the following components were added to the 1.1 μg pooled sample library, 4 μL of NEXTflex HE Universal Oligo 1, 250 μM (5′-AATGATACGGCGACCACCGAGATCTACACTCTTTCCCTACACGACGCTCTTCCGATCT-3′), 40 μL total of 25 μM NEXTflex INV-HE blocking oligos, equal volumes of each blocking oligo complementary to the barcodes in the pool (5′-CAAGCAGAAGACGGCATACGAGAT**X**GTGACT GGAGTTCAGACGTGTGCTCTTCCGATCT/C3 Spacer/-3′, where X is 8-bases of sequence specific to adapter barcode used for library construction), and 5 μL of 1 mg/mL COT-1 DNA (Invitrogen, Inc., Carlsbad, CA, USA). Samples were dried in an Eppendorf 5301 Vacuum Concentrator (Eppendorf, Hauppauge, NY, USA) at 60 °C for approximately 1 h. The dried pool was resuspended in 7.5 μL of NimbleGen Hybridization Buffer and 3.0 μL of NimbleGen Hybridization Component A, incubated at 95 °C for 10 min, and then hybridized with 4.5 μL of EZ Exome Probe Library at 47 °C for 64–72 h. Post-hybridization washing and DNA recovery were performed according to the NimbleGen SeqCap EZ Library SR Protocol.

#### Post-hybridization LM-PCR

Captured DNA pools were amplified by in LM-PCR reactions consisting of 20 μl captured library DNA, 25 μL of 2x KAPA HiFi HotStart ReadyMix, and 5 μL of 10x Library Amplification Primer Mix, containing the same two primers previously described. PCR cycling conditions were: 98 °C for 45 s, followed by 8 cycles of 98 °C for 15 s, 60 °C for 30 s, 72 °C for 30 s, with a final step of an extension at 72 °C for 1 min. Reactions were held at 4 °C until further processing. Amplified material was purified with Agencourt AMPure XP Reagent (Beckman Coulter Inc, Brea, CA, USA) in accordance with the NimbleGen SeqCap EZ Library SR Protocol. Quantification of amplified, captured libraries was performed using Kapa’s Library Quantification Kit for Illumina (Kapa Biosystems, Woburn, MA, USA) on the LightCycler 480 (Roche, Indianapolis, IN, USA).

#### Sequencing

Resulting post-capture, enriched, and multiplexed libraries were used for cluster generation on an Illumina cBOT (Illumina, San Diego, CA, USA). Paired-end sequencing (2 × 150 bp) was performed on the Illumina HiSeq 4000 following Illumina-provided protocols.

#### Detection of CH

CHIP variant calling, annotation, and filtering for somatic mutations were performed within WES data from 259 participants using Version 2.2.0 of the ArCH workflow^26^. Somatic mutations were detected using Mutect2^27^, VarDict^29^, and Lofreq^47^, whereas Pindel^48^ was used for long and complex indels. Variant annotation was performed through VEP^49^ using data from various resources, such as TOPMed^45^, MSK-IMPACT^50^, COSMIC^51^, and OncoKB^52^. Fisher’s exact tests were used to determine significantly higher mutant read depth to total depth in study participants relative to a PON (*N* = 18) composed of samples ≤60 years of age. We applied a pre-compiled list of CHIP driver genes to recover any putative variants that may have been inadvertently flagged by the recurrent filter^26^. Any germline mutations identified in the gnomAD^53^ database with an allele frequency greater than 0.0005 (0.05%) were filtered as likely germline variants. Additional filters were applied to exclude low VAF calls from Mutect2, synonymous mutations, variants exhibiting high strand bias, those with insufficient alternative allele counts, and variants located in homopolymer regions. The final analytic set included only high-confidence CHIP variants that passed all filtering criteria and were classified as putative driver mutations.

For mCAs, 110,562 participants were scanned with one of four genotyping arrays (Illumina Global Screening Array, OncoArray, Omni2.5 M, and OmniExpress). These samples met quality thresholds for harmonization and imputation^54^. mCAs were again identified using MoChA with an mCA calling threshold ≥ 0.97 and a BAF ≤ 0.03.

#### Characterization of co-occurring CH in PLCO

Following the detection of CHIP and mCAs, data were merged to determine co-occurrences. All participants with prior cancers were removed from these analyses. We then summarized the co-occurrence of CHIP and autosomal mCA events by counting the number of individuals with each possible combination. Given the limited sample size with CHIP calls, statistical evaluation of enrichment was not performed.

#### Characterization of CH in UKBB participants with prior cancer

Following the characterization of co-occurring CH in UKBB individuals with no prior cancer, similar analyses were run on UKBB participants with prior cancer. After excluding participants with non-melanoma neoplasms of the skin (*N* = 9705), this population consisted of 24,634 participants. Logistic regression models, adjusted for age, age^2^, sex, smoking status (never, former, current), and genetic similarity to reference populations, were used to compare the frequencies of specific CHIP mutations and mCAs by chromosome in both the prior and no prior cancer populations. Analyses comparing mLOX and mLOY frequencies were restricted to females and males, respectively, and sex was removed as a covariate.

#### Risk factors for CH co-occurrences

To investigate risk factors associated with CH, we used logistic regression models to evaluate associations between participant characteristics and the presence of CH in three mutually exclusive groups: (1) CHIP only, (2) autosomal mCAs only, and (3) co-occurring CHIP and autosomal mCAs. Analyses were conducted separately in TOPMed and UK Biobank participants. We then performed a random effects meta-analysis to combine results using metafor() in R (v4.4.2). The primary predictors of interest were participant characteristics, including age, sex, and smoking status. BMI was also included, but could only be investigated in UKBB participants. All sex-related estimates compared males to females. All smoking estimates compared ever smokers to never smokers. Age and BMI were treated as continuous variables, whereas sex and smoking status were treated as categorical variables. Models were adjusted for the following covariates, unless they were the variable under investigation: age, age^2^, sex, smoking status, and genetic similarity.

### Phenotypic associations in UKBB participants without prior cancer

#### Hematopoietic phenotypes

All data for hematopoietic phenotypes were extracted from the UK Biobank (Category 100081: Blood count).

We first determined whether individuals with each enriched CHIP-mCA co-occurrence had blood cell counts within normal ranges. To do this, we utilized the reference ranges quoted by the manufacturer in Table 1 of the UK Biobank Haematology Data Companion Document (https://biobank.ndph.ox.ac.uk/showcase/ukb/docs/haematology.pdf). Some parameters of interest (nucleated red blood cell count, high light scatter reticulocyte count, mean reticulocyte volume, mean sphered cell volume, platelet crit, platelet distribution width) did not have reference ranges provided and were excluded from this portion of our analyses.

All resulting blood count measures were standardized in R (v4.3.1). Multivariable linear regression adjusted for age, age^2^, sex, genetic similarity, and smoking status was used to evaluate the association between co-occurring CH groups and 22 hematopoietic-related phenotypes. Supplementary Data 21 outlines all phenotypes examined as well as the associated UK Biobank Field IDs. The Z-adjusted T/S log (UKBB Field 22191) was used within this model for associations with telomere length; full details of the measures and adjustments are found in Codd et al.^21^.

To assess the impact of co-occurring CH groups on hematopoietic phenotypes, we employed a stepwise breakdown within co-occurrence groups. The objective was twofold: first, to evaluate the effect of enriched co-occurrences, and second, to understand how the co-occurrence alters the hematopoietic landscape in comparison to individual instances of CH. By comparing the effect of co-occurrence to individual occurrences, we examined if co-occurring CH had super-additive effects on blood cell counts relative to the individual additive effects of each contributing CH.

To determine the impact of high vs. low mCA cellular fraction on hematopoietic phenotypes, some *JAK2* and *MYD88* co-occurrences (*JAK2*-chr9, *JAK2*-chr14, *MYD88*-chr3, *MYD88*-chr13, *MYD88*-chr18) were broken down into two groups. mCA cellular fraction was denoted “high” if it was ≥0.1.

To address multiple comparisons, we assessed the number of effective independent tests amongst the hematopoietic phenotypes using meff() in R (v4.3.1). 15 independent tests were determined. The significance threshold was then calculated the significance threshold by 0.05/*n*_eff_, or 0.05/15, yielding 3.33 × 10^−3^.

#### Hematological malignancy association

Using available data within the UK Biobank, we extracted cancer information from both inpatient and cancer registry data. Incident hematological cancers were defined as occurring after study enrollment. Supplementary Data 22 details hematologic disease-associated ICD-10 codes as lymphoid or myeloid cancers. We performed Cox proportional hazards regression using the coxph package in R (v4.3.1) to assess the risk of hematological malignancies (any hematological malignancy, lymphoid malignancy, myeloid malignancy) across CH groups, adjusting for age, age^2^, sex, genetic similarity, and smoking status (Never, Former, Current). Hazard ratios and 95% confidence intervals were estimated. The stepwise and cellular fraction evaluations of CH groups described in the analyses of hematopoietic phenotypes were likewise utilized within the scope of hematological malignancy associations. Co-occurrences were only stratified by cellular fraction if the resulting groups had at least 5 remaining cancer cases.

To address multiple comparisons, we determined a Bonferroni threshold of significance based on the number of co-occurrences, component events, and general CH types within the model. This calculation determined a *P* value threshold of 7.90 × 10^−4^.

#### Statistics and reproducibility

This study utilized data from large, previously established cohorts (UKBB, TOPMed studies, PLCO). Sample sizes are based on data availability within each cohort; no statistical method was used to predetermine sample size. Participants were excluded based on quality control criteria as described above. This study was based on observational data and did not involve randomization. All statistical analyses were performed in R. All statistical tests were two-sided, and multiple testing was addressed using Bonferroni correction or estimation of the effective number of independent tests, as described above. Key findings were evaluated in independent cohorts where data were available.

### Reporting summary

Further information on research design is available in the Nature Portfolio Reporting Summary linked to this article.

## Supplementary information

Supplementary Information

Transparent Peer Review file

Reporting Summary

Description of Additional Supplementary Files

Supplementary Data 1–22

## Data Availability

The data used in the present study were obtained from UKBB under application numbers 92005 and 55288 and are available upon application to the UKBB (https://www.ukbiobank.ac.uk). Data for each participating TOPMed study can be accessed through dbGaP with their corresponding TOPMed accession numbers. All genomic datasets that can be shared will be deposited in dbGaP under controlled access. Controlled access and data use restrictions are based on individual contributing TOPMed study approval. The relevant studies and dbGaP accession numbers are for each participating study are: Amish (phs000956.v5.p1) [https://www.ncbi.nlm.nih.gov/projects/gap/cgi-bin/study.cgi?study_id=phs000956.v5.p1], ARIC (phs001211.v5.p4) [https://www.ncbi.nlm.nih.gov/projects/gap/cgi-bin/study.cgi?study_id=phs001211.v5.p4], BAGS (phs001143.v4.p1) [https://www.ncbi.nlm.nih.gov/projects/gap/cgi-bin/study.cgi?study_id=phs001143.v4.p1], BioMe (phs001644.v3.p2) [https://www.ncbi.nlm.nih.gov/projects/gap/cgi-bin/study.cgi?study_id=phs001644.v3.p2], CARDIA (phs001612.v3.p3) [https://www.ncbi.nlm.nih.gov/projects/gap/cgi-bin/study.cgi?study_id=phs001612.v3.p3], CFS (phs000954.v4.p2) [https://www.ncbi.nlm.nih.gov/projects/gap/cgi-bin/study.cgi?study_id=phs000954.v4.p2], CHS (phs001368.v4.p2) [https://www.ncbi.nlm.nih.gov/projects/gap/cgi-bin/study.cgi?study_id=phs001368.v4.p2], COPDGene (phs00951.v6.p5) [https://www.ncbi.nlm.nih.gov/projects/gap/cgi-bin/study.cgi?study_id=phs000951.v6.p5], FHS (phs000974.v6.p5) [https://www.ncbi.nlm.nih.gov/projects/gap/cgi-bin/study.cgi?study_id=phs000974.v6.p5], GeneSTAR (phs001219.v3.p1) [https://www.ncbi.nlm.nih.gov/projects/gap/cgi-bin/study.cgi?study_id=phs001218.v3.p1], GENOA (phs001345.v3.p1) [https://www.ncbi.nlm.nih.gov/projects/gap/cgi-bin/study.cgi?study_id=phs001345.v3.p1], GOLDN (phs001359.v3.p1) [https://www.ncbi.nlm.nih.gov/projects/gap/cgi-bin/study.cgi?study_id=phs001359.v3.p1], HCHS/SOL (phs001395.v3.p2) [https://www.ncbi.nlm.nih.gov/projects/gap/cgi-bin/study.cgi?study_id=phs001395.v3.p2], HyperGEN (phs001293.v3.p1)[https://www.ncbi.nlm.nih.gov/projects/gap/cgi-bin/study.cgi?study_id=phs001293.v3.p1], JHS (phs000964.v5.p1) [https://www.ncbi.nlm.nih.gov/projects/gap/cgi-bin/study.cgi?study_id=phs000964.v5.p1], MESA (phs001416.v4.p1) [https://www.ncbi.nlm.nih.gov/projects/gap/cgi-bin/study.cgi?study_id=phs001416.v4.p1], VU_AF (phs001032.v6.p2) [https://www.ncbi.nlm.nih.gov/projects/gap/cgi-bin/study.cgi?study_id=phs001032.v6.p2], WGHS (phs001040.v6.p1) [https://www.ncbi.nlm.nih.gov/projects/gap/cgi-bin/study.cgi?study_id=phs001040.v6.p1], and WHI (phs001237.v4.p2) [https://www.ncbi.nlm.nih.gov/projects/gap/cgi-bin/study.cgi?study_id=phs001237.v4.p2]. For PLCO, data can be accessed through dbGaP with the following accession numbers: phs001286.v4.p2 (genotyping) [https://www.ncbi.nlm.nih.gov/projects/gap/cgi-bin/study.cgi?study_id=phs001286.v4.p2] and phs001286 (sequencing) [https://www.ncbi.nlm.nih.gov/projects/gap/cgi-bin/study.cgi?study_id=phs001286.v1.p1].

## Code availability

Mosaic chromosomal alterations were detected using MoChA pipelines (mocha.wdl), which is publicly available at 10.5281/zenodo.10892520^55^. For the most updated and detailed version, visit https://github.com/freeseek/mochawdl. All other analyses were performed using standard and previously published methods as described in the “Methods.”
