# Supplementary material for: Co-occurring clonal hematopoiesis exhibits strong selection and high leukemia risk
Source: Nat Commun. 2026 May 21;17:6682. doi: 10.1038/s41467-026-73302-x (PMC13385913; doi:10.1038/s41467-026-73302-x)
Supplement: Supplementary file 1 — Supplementary Information [file 41467_2026_73302_MOESM1_ESM.pdf]

Supplementary Information

Co-occurring clonal hematopoiesis exhibits strong selection and high leukemia risk

Kara M. Barnao, Aubrey K. Hubbard, Irenaeus C. C. Chan, Weiyin Zhou, Yasminka A. Jakubek, Giulio Genovese, Wendy S. W. Wong, Rebecca L. Kelly, Corey D. Young, Derek W. Brown, Wen-Yi Huang, Neal D. Freedman, Kristine Jones, Amy Hutchinson, Belynda Hicks, Duc Tran, Donna Arnett, Kathleen C. Barnes, Joshua C. Bis, Eric Boerwinkle, Jennifer A. Brody, April P. Carson, Daniel I. Chasman, Michael H. Cho, Pinkal Desai, Margaret F. Doyle, Myriam Fornage, Xiuqing Guo, Nancy Heard-Costa, Marguerite Ryan Irvin, Andrew D. Johnson, Sharon L. R. Kardia, Charles Kooperberg, Daniel Levy, Joshua P. Lewis, Yun Li, Ruth J. F. Loos, Taralynn M. Mack, Rasika A. Mathias, Braxton D. Mitchell, Kari E. North, Nathan Pankratz, Patricia A. Peyser, Michael H. Preuss, Bruce M. Psaty, Laura M. Raffield, Susan Redline, Stephen S. Rich, Jerome I. Rotter, Edwin K. Silverman, Albert V. Smith, Jennifer A. Smith, Adrienne Stilp, Yin Cao, Paul Scheet, Alexander P. Reiner, Alexander G. Bick, Stephen J. Chanock, Paul L. Auer, Kelly L. Bolton, Mitchell J. Machiela

I. Supplementary Figures

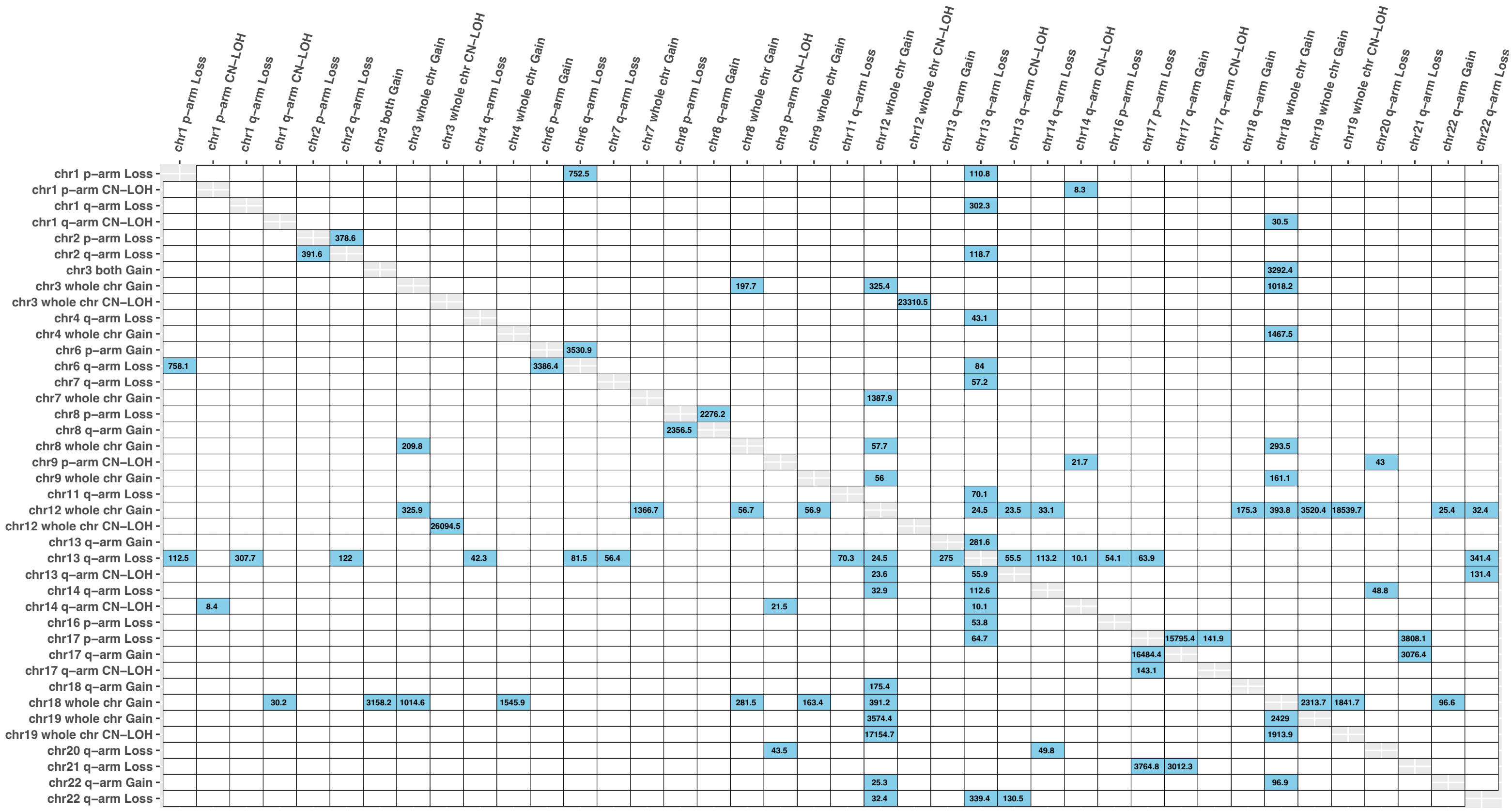

Supplementary Fig. 1. Refined analyses of enriched mCA co-occurrence in UK Biobank participants without prior cancer (N=453,807). Co-occurrence of autosomal mCAs defined by chromosomal region and event type. Associations were tested using multivariable logistic regression adjusted for age, age^2, sex, smoking status (never, former, current) and genetic similarity; odds ratios (ORs) are shown for associations with $P \leq 0.05$ (two-sided tests). Colored cells indicate Bonferroni-corrected significance ($P \leq 4.9 \times 10^{-4}$). Full results are provided in **Supplementary Data 4**.

	DNMT3A	*TET2*	*ASXL1*	*SRSF2*	*YLPM1*	*TP53*	*JAK2*	*ATM*	*MYD88*	*NFE2*
chr1 p–arm CN–LOH	2.1	2.3	3.6							
chr2 p–arm CN–LOH	8.6									
chr3 whole chr Gain		4.4							284.3	
chr4 q–arm Loss	4.1	12								
chr4 q–arm CN–LOH	2.9	66.5	14.1	34.5						
chr8 whole chr Gain	3.4						100.9			
chr9 both Gain							3962.8			
chr9 p–arm CN–LOH	1.9	6.3	5.2				1741.4			210.1
chr11 q–arm Loss			9.4							
chr11 q–arm CN–LOH		4.4						55.4		
chr12 whole chr Gain									54.3	
chr13 q–arm Loss						25.1			138.3	
chr13 q–arm CN–LOH									64.1	
chr14 q–arm CN–LOH		4.7	5.6		20.1		26.6			
chr16 q–arm Loss	4.9									
chr16 q–arm CN–LOH	2.4									
chr17 p–arm Loss						128.7				
chr17 q–arm Gain						101				
chr18 q–arm Gain									600.2	
chr18 whole chr Gain									267.8	
chr19 q–arm CN–LOH		4.6								
chr20 q–arm Loss							47.9			110.1
chr22 q–arm Loss	2.3								167.7	
chr22 q–arm CN–LOH	1.9	4.9								

Supplementary Fig. 2. Refined analyses of enriched CHIP-mCA co-occurrence in UK Biobank participants without prior cancer (N=453,807). Co-occurrence of CHIP mutations and autosomal mCAs defined by chromosomal region and event type. Associations were tested using multivariable logistic regression adjusted for age, age^2, sex, smoking status (never, former, current) and genetic similarity; odds ratios (ORs) are shown for associations with $P \leq 0.05$ (two-sided tests). Colored cells indicate Bonferroni-corrected significance ($P \leq 7.6 \times 10^{-4}$). Bold boxes

indicate the chromosomal location of each CHIP gene. Full results are provided in **Supplementary Data 6**.

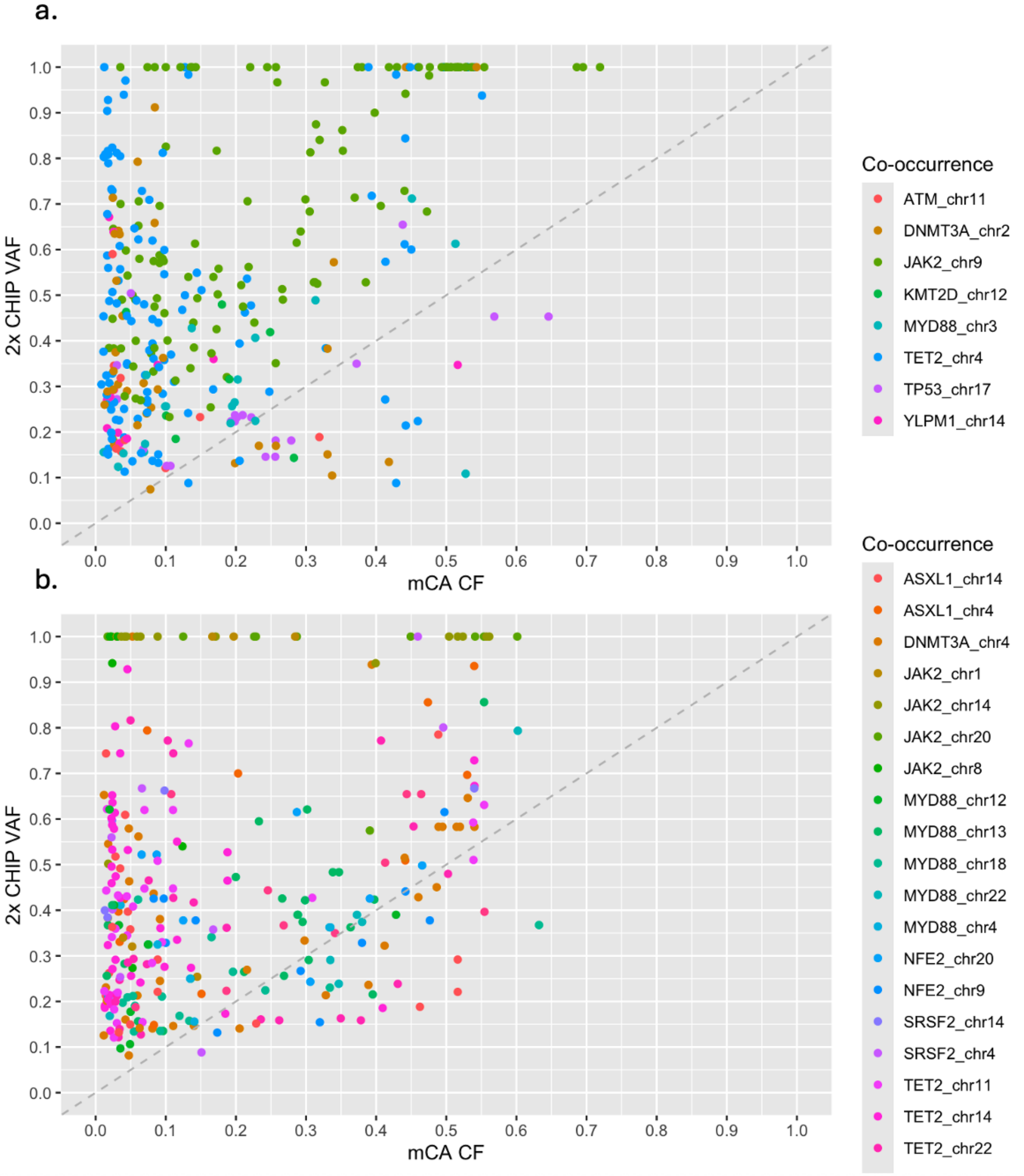

Supplementary Fig. 3. Clonal evolution of CHIP and autosomal mCA mutations in co-occurring and overlapping CH types.

Scatterplot of CHIP variant allele fraction (VAF) versus autosomal MCA cellular fraction (CF) among CHIP-mCA co-occurrences. The y-axis depicts 2× CHIP VAF and the x-axis depicts mCA CF. Each point represents a single co-occurrence; some participants have multiple events. **a,** Overlapping events: CHIP VAF exceeds mCA CF in 311/336 (92.6%) co-occurrences. **b,** Non-overlapping events: CHIP VAF exceeds mCA CF in 254/293 (86.7%) co-occurrences.

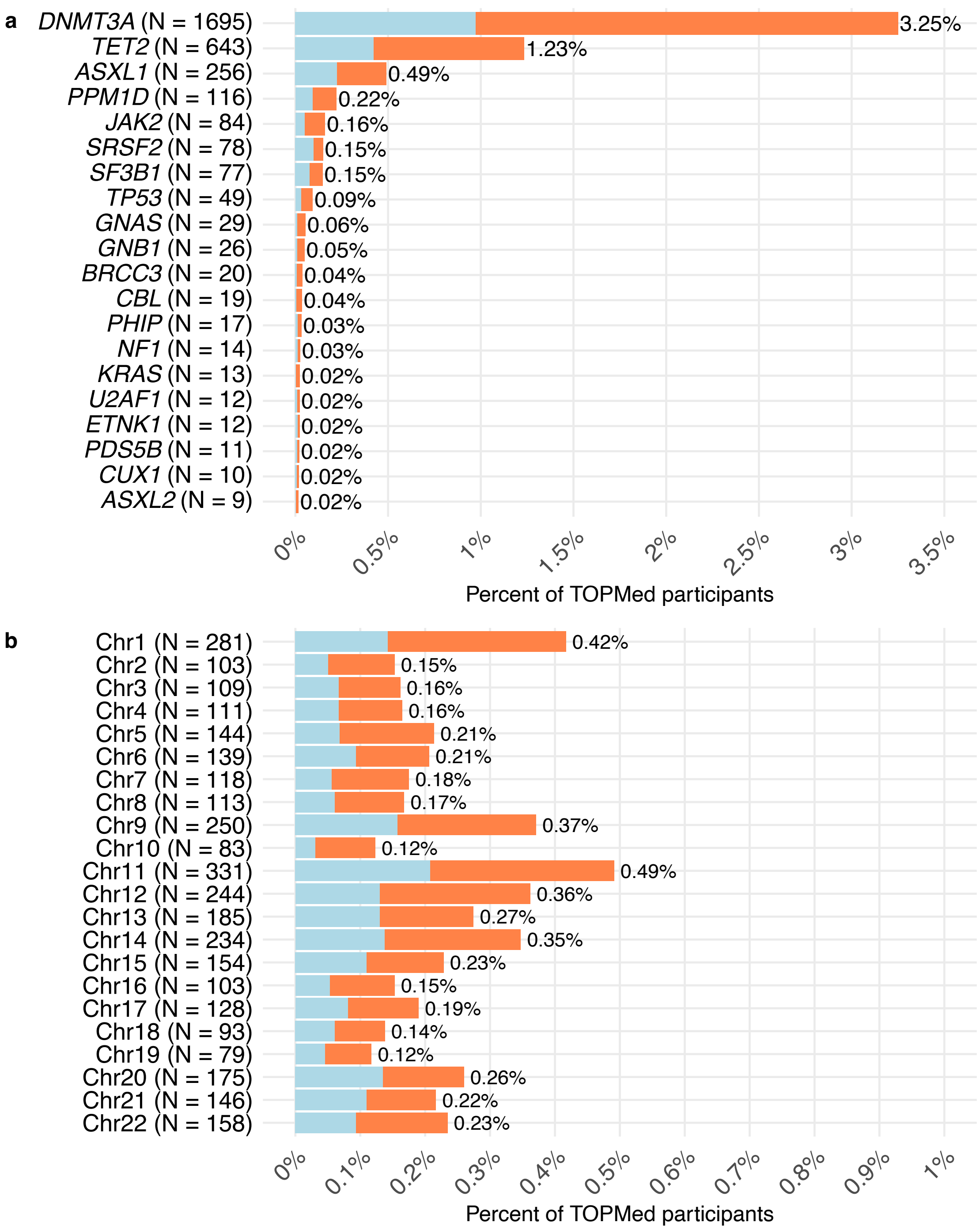

Supplementary Fig. 4. Frequencies of detectable CHIP and autosomal mCAs among TOPMed participants.a, Percentage of participants with detectable CHIP, stratified by sex female: orange, male: blue). **b,** Percentage of participants with autosomal mCAs. Percentages indicate the fraction of participants within each group (N=67,390).

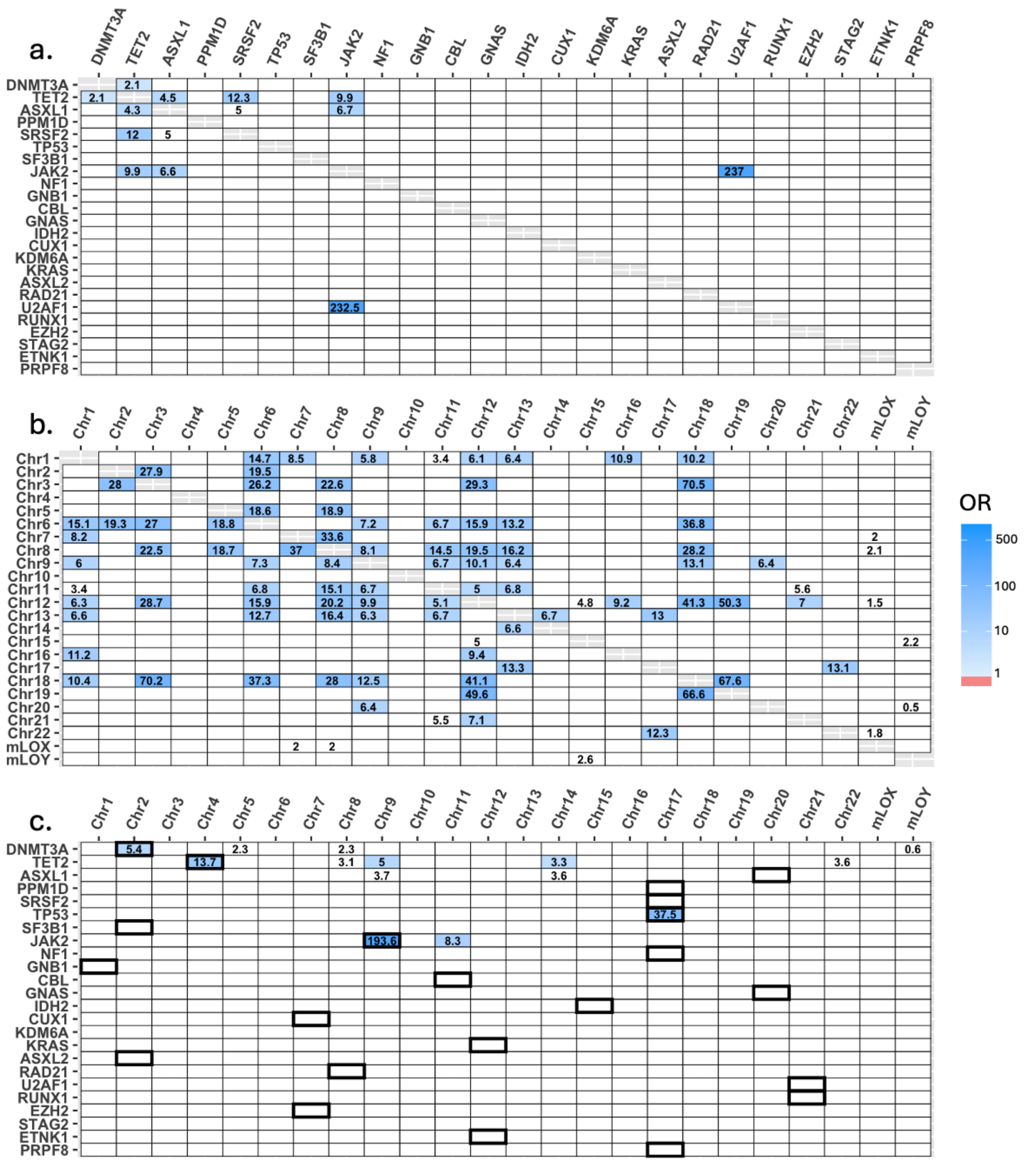

Supplementary Fig. 5. Enriched CH co-occurrences identified in TOPMed participants (N=67,390).

a, Co-occurrence of mutations in common CHIP genes. **b,** Co-occurrence of mCAs. **c,** Co-occurrence of mutations in common CHIP genes and mCAs. Associations were tested using multivariable logistic regression adjusted for age, age^2, sex, smoking status (never, ever), genetic similarity, and TOPMed study; odds ratios (ORs) are shown for associations with $P \leq 0.05$ (two-sided tests). Colored cells indicate associations that meet the Bonferroni-corrected significance threshold ($P \leq 8.7\times10^{-5}$). Bold boxes indicate the chromosomal locations of CHIP genes. Full results are provided in **Supplementary Data 7-9.**

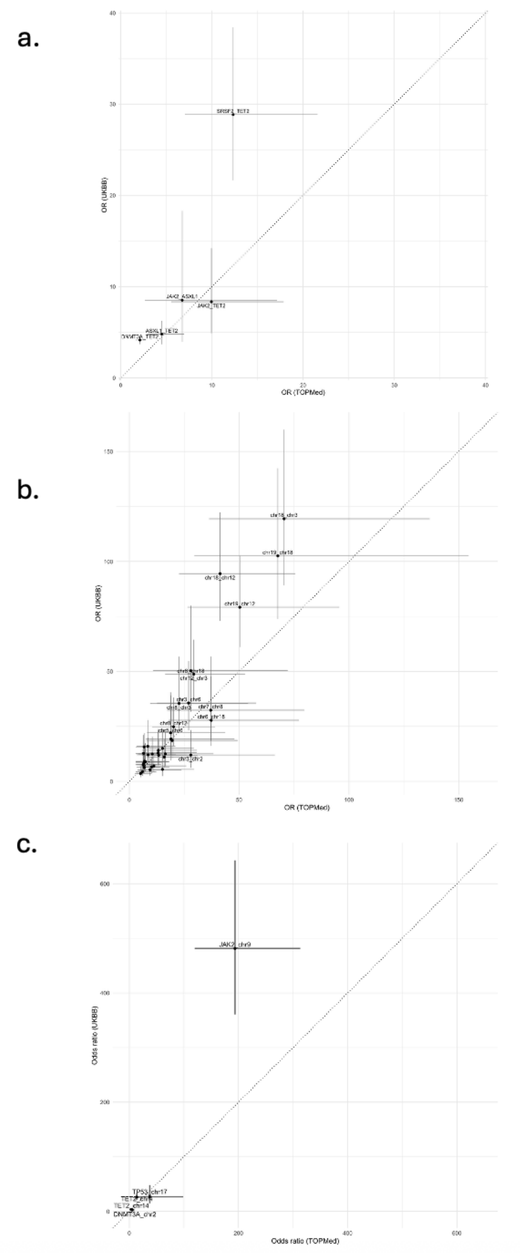

Supplementary Fig. 6. Comparison of enriched CH co-occurrences in UK Biobank and TOPMed.

Scatterplots comparing odds ratios (ORs) and 95% confidence intervals (CIs) for CH co-occurrences significantly enriched in UK Biobank participants without prior cancer (N=453,807)and TOPMed participants (N=67,390). TOPMed estimates are shown on the x-axis; UK Biobank estimates are shown on the y-axis. Points represent ORs and error bars indicate 95% CIs. Alignment near the diagonal indicates concordance in effect size across cohorts. **a,** CHIP-CHIP co-occurrences. **b,** mCA-mCA co-occurrences. **c,** CHIP-mCA co-occurrences.

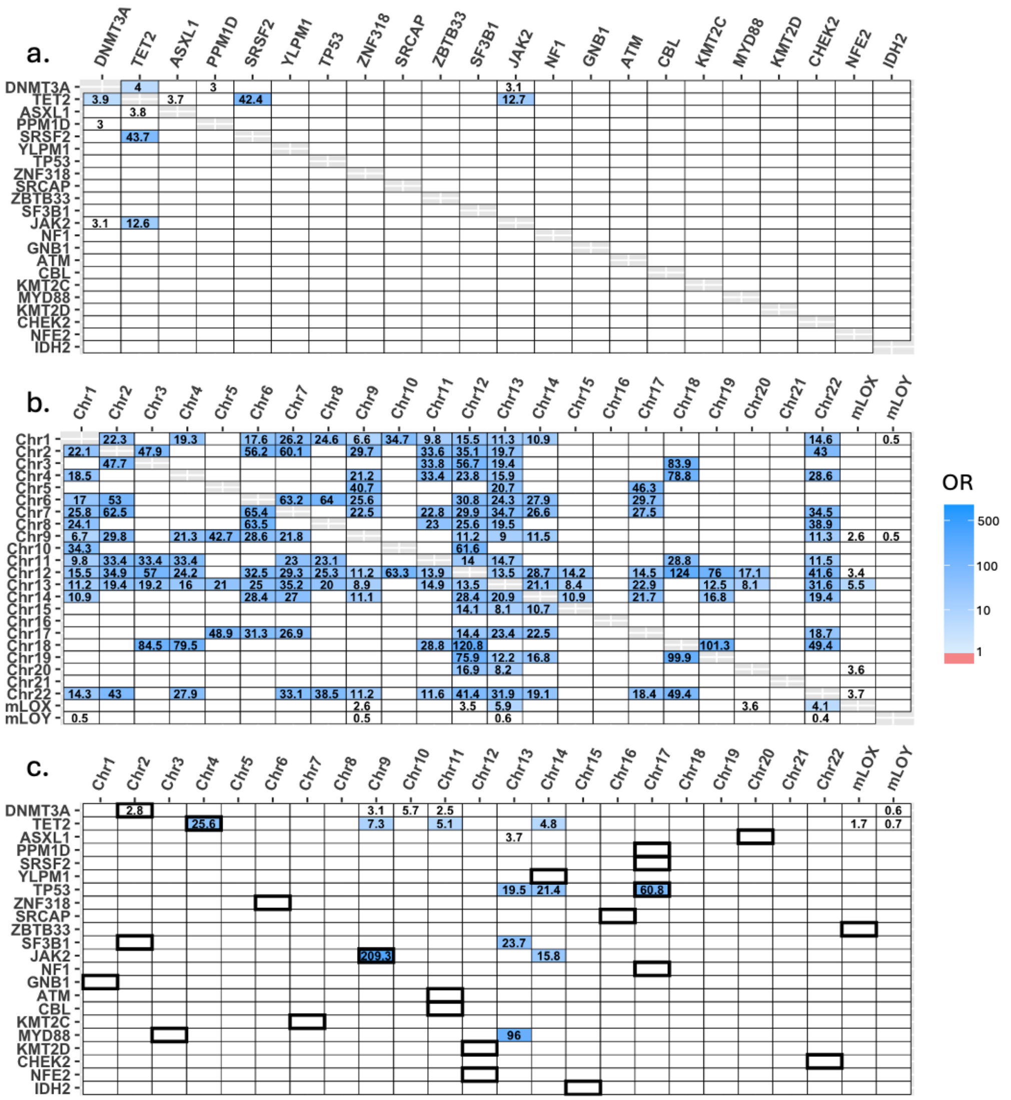

Supplementary Fig. 7. Enriched CH co-occurrences in UK Biobank participants with prior cancer at baseline (N=24,634).

a, Co-occurrence of mutations in common CHIP genes. **b,** Co-occurrence of mCAs. **c,** Co-occurrence of mutations in common CHIP genes and mCAs. Associations were tested using multivariable logistic regression adjusted for age, age^2, sex, smoking status (never, former, current), and genetic similarity; odds ratios (ORs) are shown for associations with $P \leq 0.05$ (two-sided tests). Colored cells indicate Bonferroni-corrected significance (**a,** $P \leq 2.7 \times 10^{-5}$; **b,** $P \leq 8.7 \times 10^{-5}$; **c,** $P \leq 4.8 \times 10^{-5}$). Bold boxes indicate chromosomal locations of CHIP genes. Full results are provided in **Supplementary Data 13-15**.

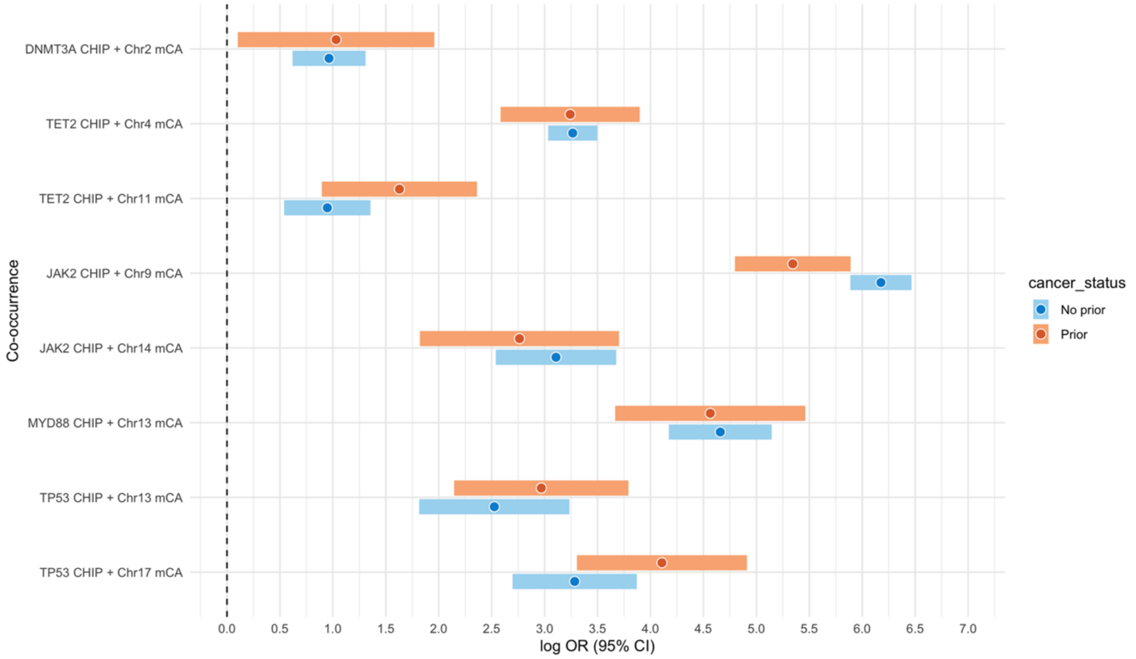

Supplementary Fig. 8. Comparison of enriched CHIP-autosomal mCA co-occurrences in UK Biobank participants with and without prior cancer.

Forest plot showing log-transformed odds ratios (ORs) and 95% confidence intervals (CIs) for CHIP-mCA co-occurrences that were significantly enriched (two-sided tests) in participants with (orange; N=24,634) and without (blue; N=453,807) prior cancer at baseline. Points (circles) show ORs and error bars indicate 95% CIs derived from multivariable logistic regression models adjusted for age, age^2, sex, smoking status, and genetic similarity. Full results are provided in **Supplementary Data 5** (without prior cancer) and **Supplementary Data 15** (prior cancer).

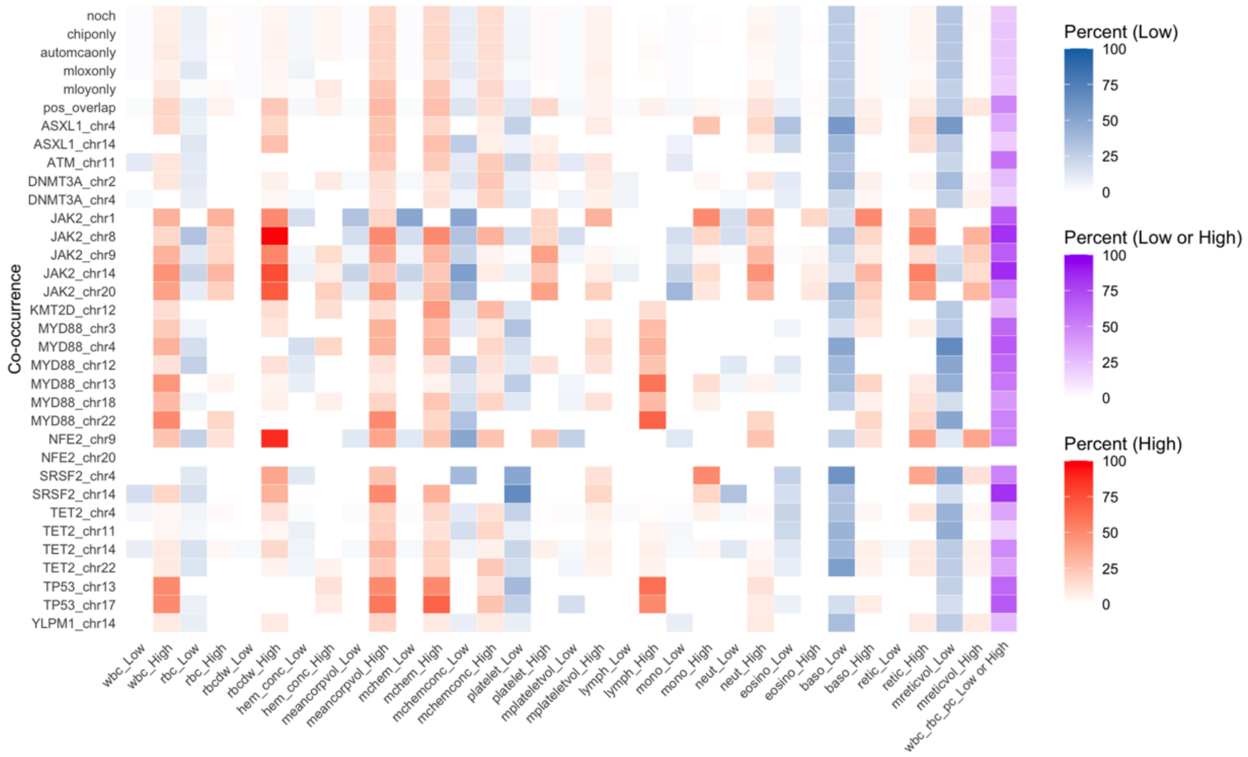

Supplementary Fig. 9. Frequencies of abnormal blood cell measurements across CH types and co-occurrences.

Heatmap showing the percentage of individuals (N=453,807) with each CH type who have blood cell measurements below (blue) or above (red) reference ranges defined in the UK Biobank Haematology Data Companion Document. The final column (purple) shows the percentage of any abnormal white blood cell, red blood cell, or platelet count. Sample size varies by CH type. The traits are shortened as follows: wbc = white blood cell count; rbc = red blood cell count; rbcdw = red blood cell distribution width; hem_conc = hemoglobin concentration; meancorpvol = mean corpuscular volume; mchem = mean corpuscular hemoglobin; mchemconc = mean corpuscular hemoglobin concentration; platelet = platelet count; mplateletvol = mean platelet volume; lymph = lymphocyte count; mono = monocyte count; neut = neutrophil count; eosino = eosinophil count; baso = basophil count; retic = reticulocyte count; mreticvol = mean reticulocyte volume.

II. Supplementary Acknowledgements

Genetics of Cardiometabolic Health in the Amish **(AMISH)**

The TOPMed component of the Amish Research Program was supported by NIH grants R01 HL121007, U01 HL072515, and R01 AG18728.

Email Braxton Mitchell (bmitchel@som.umaryland.edu) for additional input.

Atherosclerosis Risk in Communities Study VTE cohort **(ARIC)**

The Atherosclerosis Risk in Communities study has been funded in whole or in part with Federal funds from the National Heart, Lung, and Blood Institute, National Institutes of Health, Department of Health and Human Services under Contract nos. (75N92022D00001, 75N92022D00002, 75N92022D00003, 75N92022D00004, 75N92022D00005). The authors thank the staff and participants of the ARIC study for their important contributions.

Barbados Asthma Genetics Study **(BAGS)**

The Genetics and Epidemiology of Asthma in Barbados is supported by National Institutes of Health (NIH) National Heart, Lung, Blood Institute TOPMed (R01 HL104608-S1) and: R01 AI20059, K23 HL076322, and RC2 HL101651.

Authorized access to genotype data may be obtained through accession number phs001143.
For the specific cohort descriptions and descriptions regarding the collection of phenotype data can be found at:
https://topmed.nhlbi.nih.gov/group/bags-asthma .

The authors wish to give special recognition to the individual study participants who provided biological samples and or data, without their support in research none of this would be possible.

Mount Sinai BioMe Biobank **(BioMe)**

The Mount Sinai BioMe Biobank has been supported by The Andrea and Charles Bronfman Philanthropies and in part by Federal funds from the NHLBI and NHGRI (U01HG00638001; U01HG007417; X01HL134588). We thank all participants in the Mount Sinai Biobank. We also thank all our recruiters who have assisted and continue to assist in data collection and management and are grateful for the computational resources and staff expertise provided by Scientific Computing at the Icahn School of Medicine at Mount Sinai.

Coronary Artery Risk Development in Young Adults **(CARDIA)**

The Coronary Artery Risk Development in Young Adults Study (CARDIA) is conducted and supported by the National Heart, Lung, and Blood Institute (NHLBI) in collaboration with the University of Alabama at Birmingham (HHSN268201800005I & HHSN268201800007I), Northwestern University (HHSN268201800003I), University of Minnesota

(HHSN268201800006I), and Kaiser Foundation Research Institute (HHSN268201800004I). CARDIA was also partially supported by the Intramural Research Program of the National Institute on Aging (NIA) and an intra-agency agreement between NIA and NHLBI (AG0005).

Molecular data for the Trans-Omics in Precision Medicine (TOPMed) program was supported by the National Heart, Lung and Blood Institute (NHLBI). Genome Sequencing for "NHLBI TOPMed: Coronary Artery Risk Development in Young Adults" (phs001612.v3.p3) was performed at Baylor College of Medicine Human Genome Sequencing Center (HHSN268201600033I). Core support including centralized genomic read mapping and genotype calling, along with variant quality metrics and filtering were provided by the TOPMed Informatics Research Center (3R01HL-117626-02S1; contract HHSN268201800002I). Core support including phenotype harmonization, data management, sample-identity QC, and general program coordination were provided by the TOPMed Data Coordinating Center (R01HL-120393; U01HL-120393; contract HHSN268201800001I). We gratefully acknowledge the studies and participants who provided biological samples and data for TOPMed.

Cleveland Family Study **(CFS)**

The Cleveland Family Study has been supported in part by National Institutes of Health grants [R01-HL046380, KL2-RR024990, R35-HL135818, and R01-HL113338].

Molecular data for the Trans-Omics in Precision Medicine (TOPMed) program was supported by the National Heart, Lung and Blood Institute (NHLBI). Genome Sequencing for "NHLBI TOPMed: Cleveland Family Study" (phs000954.v4.p2) was performed at Northwest Genomics Center (3R01HL098433-05S1). Core support including centralized genomic read mapping and genotype calling, along with variant quality metrics and filtering were provided by the TOPMed Informatics Research Center (3R01HL-117626-02S1; contract HHSN268201800002I). Core support including phenotype harmonization, data management, sample-identity QC, and general program coordination were provided by the TOPMed Data Coordinating Center (R01HL-120393; U01HL-120393; contract HHSN268201800001I). We gratefully acknowledge the studies and participants who provided biological samples and data for TOPMed.

Cardiovascular Health Study **(CHS)**

This research was supported by contracts HHSN268201200036C, HHSN268200800007C, HHSN268201800001C, N01HC55222, N01HC85079, N01HC85080, N01HC85081, N01HC85082, N01HC85083, N01HC85086, 75N92021D00006, and grants U01HL080295, U01HL130114, R01HL172803, and R01HL105756 from the National Heart, Lung, and Blood Institute (NHLBI), with additional contribution from the National Institute of Neurological Disorders and Stroke (NINDS). Additional support was provided by R01AG023629 from the National Institute on Aging (NIA). A full list of principal CHS investigators and institutions can be found at CHS-NHLBI.org.

The content is solely the responsibility of the authors and does not necessarily represent the official views of the National Institutes of Health.

Genetic Epidemiology of COPD Study **(COPDGene)**

The COPDGene study (NCT00608764) is supported by grants from the NHLBI (U01HL089897 and U01HL089856), by NIH contract 75N92023D00011, and by the COPD Foundation through contributions made to an Industry Advisory Committee that has included AstraZeneca, Bayer Pharmaceuticals, Boehringer-Ingelheim, Genentech, GlaxoSmithKline, Novartis, Pfizer and Sunovion.

A full listing of COPDGene investigators can be found at: http://www.copdgene.org/directory.

Framingham Heart Study **(FHS)**

The Framingham Heart Study (FHS) acknowledges the support of contracts NO1-HC-25195, HHSN268201500001I, 75N92019D00031 and 75N92025D00012 from the National Heart, Lung and Blood Institute, grant supplement R01 HL092577-06S1, and TOPMed X01 HL139389 for this research. We also acknowledge the dedication of the FHS study participants without whom this research would not be possible.

Genetic Studies of Atherosclerosis Risk **(GeneSTAR)**

GeneSTAR was supported by the National Institutes of Health/National Heart, Lung, and Blood Institute (U01 HL72518, HL087698, HL112064, HL11006, HL118356) and by a grant from the National Institutes of Health/National Center for Research Resources (M01-RR000052) to the Johns Hopkins General Clinical Research Center. We would like to thank our participants and staff for their valuable contributions.

Molecular data for the Trans-Omics in Precision Medicine (TOPMed) program was supported by the National Heart, Lung and Blood Institute (NHLBI). Genome Sequencing for "NHLBI TOPMed: Genetic Studies of Atherosclerosis Risk" (phs001219.v3.p1) was performed at Illumina (R01HL112064), Psomagen (formerly Macrogen; 3R01HL112064-04S1), and the Broad Institute (HHSN268201500014C). Core support including centralized genomic read mapping and genotype calling, along with variant quality metrics and filtering were provided by the TOPMed Informatics Research Center (3R01HL-117626-02S1; contract HHSN268201800002I). Core support including phenotype harmonization, data management, sample-identity QC, and general program coordination were provided by the TOPMed Data Coordinating Center (R01HL-120393; U01HL-120393; contract HHSN268201800001I). We gratefully acknowledge the studies and participants who provided biological samples and data for TOPMed.

Genetic Epidemiology Network of Arteriopathy **(GENOA)**

Support for GENOA was provided by the National Heart, Lung and Blood Institute (U01HL054457, U01HL054464, U01HL054481, R01HL119443, and R01HL087660) of the National Institutes of Health. We would like the thank the Mayo Clinic Genotyping Core, the DNA Sequencing and Gene Analysis Center at the University of Washington, and the Broad Institute for their genotyping and sequencing services. We would also like to thank the GENOA participants.

Molecular data for the Trans-Omics in Precision Medicine (TOPMed) program was supported by the National Heart, Lung and Blood Institute (NHLBI). Genome Sequencing for "NHLBI TOPMed: Genetic Epidemiology Network of Arteriopathy" (phs001345.v3.p1) was performed at Northwest Genomics Center (3R01HL055673-18S1). Core support including centralized genomic read mapping and genotype calling, along with variant quality metrics and filtering were provided by the TOPMed Informatics Research Center (3R01HL-117626-02S1; contract HHSN268201800002I). Core support including phenotype harmonization, data management, sample-identity QC, and general program coordination were provided by the TOPMed Data Coordinating Center (R01HL-120393; U01HL-120393; contract HHSN268201800001I). We gratefully acknowledge the studies and participants who provided biological samples and data for TOPMed.

Genetics of Lipid Lowering Drugs and Diet Network **(GOLDN)**

GOLDN biospecimens, baseline phenotype data, and intervention phenotype data were collected with funding from National Heart, Lung and Blood Institute (NHLBI) grant U01 HL072524. Whole-genome sequencing in GOLDN was funded by NHLBI grant R01 HL104135 and supplement R01 HL104135-04S1. We are grateful to the staff of the GOLDN study for assistance in data/ sample collection and management.

Molecular data for the Trans-Omics in Precision Medicine (TOPMed) program was supported by the National Heart, Lung and Blood Institute (NHLBI). Genome Sequencing for "NHLBI TOPMed: Hispanic Community Health Study/Study of Latinos" (phs001395.v3.p2) was performed at Northwest Genomics Center (3R01HL104135-04S1). Core support including centralized genomic read mapping and genotype calling, along with variant quality metrics and filtering were provided by the TOPMed Informatics Research Center (3R01HL-117626-02S1; contract HHSN268201800002I). Core support including phenotype harmonization, data management, sample-identity QC, and general program coordination were provided by the TOPMed Data Coordinating Center (R01HL-120393; U01HL-120393; contract HHSN268201800001I). We gratefully acknowledge the studies and participants who provided biological samples and data for TOPMed.

Hispanic Community Health Study- Study of Latinos **(HCHS_SOL)**

The Hispanic Community Health Study/Study of Latinos is a collaborative study supported by contracts from the National Heart, Lung, and Blood Institute (NHLBI) to the University of North Carolina (HHSN268201300001I / N01-HC-65233), University of Miami (HHSN268201300004I / N01-HC-65234), Albert Einstein College of Medicine (HHSN268201300002I / N01-HC-65235), University of Illinois at Chicago – HHSN268201300003I / N01-HC-65236 Northwestern Univ), and San Diego State University (HHSN268201300005I / N01-HC-65237). The following Institutes/Centers/Offices have contributed to the HCHS/SOL through a transfer of funds to the NHLBI: National Institute on Minority Health and Health Disparities, National Institute on Deafness and Other Communication Disorders, National Institute of Dental and Craniofacial Research, National Institute of Diabetes and Digestive and Kidney Diseases, National Institute of Neurological Disorders and Stroke, NIH Institution-Office of Dietary Supplements. The authors thank the staff and participants of the HCHS/ SOL Study for their important contributions.

Molecular data for the Trans-Omics in Precision Medicine (TOPMed) program was supported by the National Heart, Lung and Blood Institute (NHLBI). Genome Sequencing for "NHLBI TOPMed: Hispanic Community Health Study/Study of Latinos" (phs001395.v3.p2) was performed at Baylor College of Medicine Human Genome Sequencing Center (HHSN268201600033I). Core support including centralized genomic read mapping and genotype calling, along with variant quality metrics and filtering were provided by the TOPMed Informatics Research Center (3R01HL-117626-02S1; contract HHSN268201800002I). Core support including phenotype harmonization, data management, sample-identity QC, and general program coordination were provided by the TOPMed Data Coordinating Center (R01HL-120393; U01HL-120393; contract HHSN268201800001I). We gratefully acknowledge the studies and participants who provided biological samples and data for TOPMed.

Hypertension Genetic Epidemiology Network (**HyperGEN)**

The HyperGEN Study is part of the National Heart, Lung, and Blood Institute (NHLBI) Family Blood Pressure Program; collection of the data represented here was supported by grants U01 HL054472 (MN Lab), U01 HL054473 (DCC), U01 HL054495 (AL FC), and U01 HL054509 (NC FC). The HyperGEN: Genetics of Left Ventricular Hypertrophy Study was supported by NHLBI grant R01 HL055673 with whole-genome sequencing made possible by supplement -18S1. We thank the participants and investigators of the HyperGEN: Genetics of Left Ventricular Hypertrophy Study and its TOPMed supplement (R01HL055673, 3R01HL055673-18S1) for their generous contributions to this study.

Jackson Heart Study **(JHS)**

The Jackson Heart Study (JHS) is supported and conducted in collaboration with Jackson State University (HHSN268201800013I), Tougaloo College (HHSN268201800014I), the Mississippi State Department of Health (HHSN268201800015I/HHSN26800001) and the University of Mississippi Medical Center (HHSN268201800010I, HHSN268201800011I and

HHSN268201800012I) contracts from the National Heart, Lung, and Blood Institute (NHLBI) and the National Institute for Minority Health and Health Disparities (NIMHD). The authors also wish to thank the staffs and participants of the JHS.

Multi-Ethnic Study of Atherosclerosis **(MESA)**

Whole genome sequencing (WGS) for the Trans-Omics in Precision Medicine (TOPMed) program was supported by the National Heart, Lung and Blood Institute (NHLBI). WGS for "NHLBI TOPMed: Multi-Ethnic Study of Atherosclerosis (MESA)" (phs001416.v1.p1) was performed at the Broad Institute of MIT and Harvard (3U54HG003067-13S1). Centralized read mapping and genotype calling, along with variant quality metrics and filtering were provided by the TOPMed Informatics Research Center (3R01HL-117626-02S1). Phenotype harmonization, data management, sample-identity QC, and general study coordination, were provided by the TOPMed Data Coordinating Center (3R01HL-120393-02S1). The MESA projects are conducted and supported by the National Heart, Lung, and Blood Institute (NHLBI) in collaboration with MESA investigators. Support for MESA is provided by contracts 75N92025D00022, 75N92020D00001, HHSN268201500003I, N01-HC-95159, 75N92025D00026, 75N92020D00005, N01-HC-95160, 75N92020D00002, N01-HC-95161, 75N92025D00024, 75N92020D00003, N01-HC-95162, 75N92025D00027, 75N92020D00006, N01-HC-95163, 75N92025D00025, 75N92020D00004, N01-HC-95164, 75N92025D00028, 75N92020D00007, N01-HC-95165, N01-HC-95166, N01-HC-95167, N01-HC-95168, N01-HC-95169, UL1-TR-000040, UL1-TR-001079, UL1-TR-001420, UL1TR001881, and R01HL105756. The authors thank the MESA participants and the MESA investigators and staff for their valuable contributions. A full list of participating MESA investigators and institutions can be found at http://www.mesa-nhlbi.org.

The Vanderbilt Atrial Fibrillation Registry **(VU_AF)**

The research reported in this article was supported by grants from the American Heart Association to Dr. Darbar (EIA 0940116N), and grants from the National Institutes of Health (NIH) to Dr. Darbar (HL092217), and Dr. Roden (U19 HL65962, and UL1 RR024975). This project was also supported by CTSA award (UL1TR000445) from the National Center for Advancing Translational Sciences. Its contents are solely the responsibility of the authors and do not necessarily represent official views of the National Center for Advancing Translational Sciences of the NIH.

Women's Genome Health Study **(WGHS)**

The WGHS is supported by the National Heart, Lung, and Blood Institute (HL043851 and HL080467) and the National Cancer Institute (CA047988 and UM1CA182913). The most recent cardiovascular endpoints were supported by ARRA funding HL099355.

Women's Health Initiative **(WHI)**

The WHI program is funded by the National Heart, Lung, and Blood Institute, National Institutes of Health, U.S. Department of Health and Human Services through contracts 75N92021D00001, 75N92021D00002, 75N92021D00003, 75N92021D00004, 75N92021D00005.
