# Supplementary material for: Co-occurring clonal hematopoiesis exhibits strong selection and high leukemia risk
Source: Nat Commun. 2026 May 21;17:6682. doi: 10.1038/s41467-026-73302-x (PMC13385913; doi:10.1038/s41467-026-73302-x)
Supplement: Supplementary file 3 — Reporting Summary [file 41467_2026_73302_MOESM3_ESM.pdf]

nature portfolio

Corresponding author(s): Kara M. Barnao, Mitchell J. Machiela

Last updated by author(s): 03/30/2026

Reporting Summary

Nature Portfolio wishes to improve the reproducibility of the work that we publish. This form provides structure for consistency and transparency in reporting. For further information on Nature Portfolio policies, see our Editorial Policies and the Editorial Policy Checklist.

Statistics

For all statistical analyses, confirm that the following items are present in the figure legend, table legend, main text, or Methods section.

n/a	Confirmed	
☐	☒	The exact sample size (*n*) for each experimental group/condition, given as a discrete number and unit of measurement
☐	☒	A statement on whether measurements were taken from distinct samples or whether the same sample was measured repeatedly
☐	☒	The statistical test(s) used AND whether they are one- or two-sided<br>*Only common tests should be described solely by name; describe more complex techniques in the Methods section.*
☐	☒	A description of all covariates tested
☐	☒	A description of any assumptions or corrections, such as tests of normality and adjustment for multiple comparisons
☐	☒	A full description of the statistical parameters including central tendency (e.g. means) or other basic estimates (e.g. regression coefficient) AND variation (e.g. standard deviation) or associated estimates of uncertainty (e.g. confidence intervals)
☐	☒	For null hypothesis testing, the test statistic (e.g. *F*, *t*, *r*) with confidence intervals, effect sizes, degrees of freedom and *P* value noted<br>*Give P values as exact values whenever suitable.*
☒	☐	For Bayesian analysis, information on the choice of priors and Markov chain Monte Carlo settings
☒	☐	For hierarchical and complex designs, identification of the appropriate level for tests and full reporting of outcomes
☐	☒	Estimates of effect sizes (e.g. Cohen's *d*, Pearson's *r*), indicating how they were calculated

Our web collection on statistics for biologists contains articles on many of the points above.

Software and code

Policy information about availability of computer code

Data collection	No software was used for data collection.
Data analysis	The following tools were used for data analysis: ArCH (v2.2.0), Mutect2 (v4.2.1.0), VarDict (v1.6.0), MoChA (https://github.com/freeseek/mochawdl), Lofreq, Pindel, R (v4.3.1, v.4.4.2), GATK's BQSRPipelineSpark (v4.2.0.0), SNPweights, GrafPop, and Ensembl Variant Effect Predictor.

For manuscripts utilizing custom algorithms or software that are central to the research but not yet described in published literature, software must be made available to editors and reviewers. We strongly encourage code deposition in a community repository (e.g. GitHub). See the Nature Portfolio guidelines for submitting code & software for further information.

Data

Policy information about availability of data

All manuscripts must include a data availability statement. This statement should provide the following information, where applicable:

- Accession codes, unique identifiers, or web links for publicly available datasets
- A description of any restrictions on data availability
- For clinical datasets or third party data, please ensure that the statement adheres to our policy

The data used in the present study were obtained from UK Biobank (UKBB) under application numbers 92005 and 55288, and are available upon application to the UKBB (https://www.ukbiobank.ac.uk).
Data for each participating TOPMed study can be accessed through dbGaP with their corresponding TOPMed accession numbers. All genomic datasets that can be

shared will be deposited in dbGaP under controlled access. Controlled access and data use restrictions are based on individual contributing TOPMed study approval. The relevant studies and dbGaP accession numbers are for each participating study are: Amish (phs000956.v5.p1), ARIC (phs001211.v5.p4), BAGS (phs001143.v4.p1), BioMe (phs001644.v3.p2), CARDIA (phs001612.v3.p3), CFS (phs000954.v4.p2), CHS (phs001368.v4.p2), COPDGene (phs00951.v6.p5), FHS (phs000974.v6.p5), GeneSTAR (phs001219.v3.p1), GENOA (phs001345.v3.p1), GOLDN (phs001359.v3.p1), HCHS/SOL (phs001395.v3.p2), HyperGEN (phs001293.v3.p1), JHS (phs000964.v5.p1), MESA (phs001416.v4.p1), VU_AF (phs001032.v6.p2), WGHS (phs001040.v6.p1), and WHI (phs001237.v4.p2).

For PLCO, data can be accessed through dbGaP with the following accession number: phs001286.v1.p1.

The data used in the present study were obtained from UK Biobank (UKBB) under application numbers 92005 and 55288, and are available upon application to the UKBB (https://www.ukbiobank.ac.uk).

Data for each participating TOPMed study can be accessed through dbGaP with their corresponding TOPMed accession numbers. All genomic datasets that can be shared will be deposited in dbGaP under controlled access. Controlled access and data use restrictions are based on individual contributing TOPMed study approval. The relevant studies and dbGaP accession numbers are for each participating study are: Amish (phs000956.v5.p1), ARIC (phs001211.v5.p4), BAGS (phs001143.v4.p1), BioMe (phs001644.v3.p2), CARDIA (phs001612.v3.p3), CFS (phs000954.v4.p2), CHS (phs001368.v4.p2), COPDGene (phs00951.v6.p5), FHS (phs000974.v6.p5), GeneSTAR (phs001219.v3.p1), GENOA (phs001345.v3.p1), GOLDN (phs001359.v3.p1), HCHS/SOL (phs001395.v3.p2), HyperGEN (phs001293.v3.p1), JHS (phs000964.v5.p1), MESA (phs001416.v4.p1), VU_AF (phs001032.v6.p2), WGHS (phs001040.v6.p1), and WHI (phs001237.v4.p2).

For PLCO, data can be accessed through dbGaP with the following accession number: phs001286.v1.p1.

Research involving human participants, their data, or biological material

Policy information about studies with human participants or human data. See also policy information about sex, gender (identity/presentation), and sexual orientation and race, ethnicity and racism.

Reporting on sex and gender	Sex was self-reported and confirmed using genetic markers.
Reporting on race, ethnicity, or other socially relevant groupings	Genetic similarity to 1000 Genomes reference populations (CEU, YRI, ASN) was estimated in SNPweights for UKBB participants. For TOPMed, genetic similarity proportions for the percentage of African, Asian, and European ancestry were inferred for each participant using GrafPop.
Population characteristics	The study population consists of male and female participants from large biobanks that contributed blood samples. Median age ranged from 58.2 (sd = 8.1) for UKBB to 67 (sd = 5.3) for PLCO.
Recruitment	Recruitment for each contributing study varied by study objectives. For the UKBB, all men and women between 40–69 years of age who were registered with the National Health Service and lived within approximately 25 miles of one of the 22 study assessment centers were invited to participate between 2006 and 2010. In total, approximately 9.2 million invitations were sent, resulting in the recruitment of 503,325 participants (a response rate of 5.5%).<br><br>For contributing TOPMed studies, recruitment aimed to enroll participants reflective of the study target population. Additional details on recruitment can be found in the study-specific references provided.<br><br>For PLCO, participants between the ages of 55 and 74 were recruited between 1993 and 2001 at 10 different assessment centers. Participants had no known history of prostate, lung, colorectal, or ovarian cancer.
Ethics oversight	All studies received institutional review board (IRB) approval. Informed consent was obtained from all participants.

Note that full information on the approval of the study protocol must also be provided in the manuscript.

Field-specific reporting

Please select the one below that is the best fit for your research. If you are not sure, read the appropriate sections before making your selection.

[x] Life sciences [ ] Behavioural & social sciences [ ] Ecological, evolutionary & environmental sciences

For a reference copy of the document with all sections, see nature.com/documents/nr-reporting-summary-flat.pdf

Life sciences study design

All studies must disclose on these points even when the disclosure is negative.

Sample size	The total combined sample size for this study was 546,090 participants. Sample size was not determined by statistical power calculations; instead, it was predetermined by data availability from three biobanks (UK Biobank, TOPMed, PLCO). Samples were recruited from these studies with existing whole-exome or whole-genome sequencing and genotyping array data for calling CH and for which data use permissions permitted the investigation of clonal hematopoiesis (CH). The use of these cohorts provides substantial statistical power to detect CH co-occurrence and associations, including for relatively rare events, and is consistent with established practices in population-scale genomic studies.
Data exclusions	Individuals who failed genotyping or sequencing quality control or exhibited sex discordance were removed.
Replication	Reproducibility was assessed by replicating key analyses in independent cohorts. Initial findings of enriched CH co-occurrences identified in the YK Biobank (N=478,441) were successfully replicated in TOPMed (N=67,390) and in a subset of PLCO participants with mCAs (N=259). However, analyses examining associations between CH co-occurrence and various phenotypes could not be replicated due to incomplete data in the replication cohorts.

Randomization	This study did not incorporate randomization as it was observational in design and did not involve assignment of participants with intervention or exposure conditions. Exposures were not controlled by the investigators in this study. As such, random assignment was neither feasible nor appropriate. To address possible confounding, known and suspected covariates were controlled for through statistical adjustment in multivariable linear and logistic regression and Cox proportional hazards models.
Blinding	Blinding was not performed given that this was an observational study with no investigator-controlled intervention. Therefore, there was no risk of bias that blinding procedures intend to reduce.

Reporting for specific materials, systems and methods

We require information from authors about some types of materials, experimental systems and methods used in many studies. Here, indicate whether each material, system or method listed is relevant to your study. If you are not sure if a list item applies to your research, read the appropriate section before selecting a response.

Materials & experimental systems

n/a	Involved in the study
☒	☐ Antibodies
☒	☐ Eukaryotic cell lines
☒	☐ Palaeontology and archaeology
☒	☐ Animals and other organisms
☒	☐ Clinical data
☒	☐ Dual use research of concern
☒	☐ Plants

Methods

n/a	Involved in the study
☒	☐ ChIP-seq
☒	☐ Flow cytometry
☒	☐ MRI-based neuroimaging

Plants

Seed stocks	*Report on the source of all seed stocks or other plant material used. If applicable, state the seed stock centre and catalogue number. If plant specimens were collected from the field, describe the collection location, date and sampling procedures.*
Novel plant genotypes	*Describe the methods by which all novel plant genotypes were produced. This includes those generated by transgenic approaches, gene editing, chemical/radiation-based mutagenesis and hybridization. For transgenic lines, describe the transformation method, the number of independent lines analyzed and the generation upon which experiments were performed. For gene-edited lines, describe the editor used, the endogenous sequence targeted for editing, the targeting guide RNA sequence (if applicable) and how the editor was applied.*
Authentication	*Describe any authentication procedures for each seed stock used or novel genotype generated. Describe any experiments used to assess the effect of a mutation and, where applicable, how potential secondary effects (e.g. second site T-DNA insertions, mosiacism, off-target gene editing) were examined.*
