# Supplementary material for: Co-occurring clonal hematopoiesis exhibits strong selection and high leukemia risk
Source: Nat Commun. 2026 May 21;17:6682. doi: 10.1038/s41467-026-73302-x (PMC13385913; doi:10.1038/s41467-026-73302-x)
Supplement: Supplementary file 4 — Description of Additional Supplementary Files [file 41467_2026_73302_MOESM4_ESM.pdf]

Description of Additional Supplementary Files

Supplementary Data 1. Demographic summary measures of UK Biobank participants with no prior cancer diagnosis. Participants may be accounted for multiple times within CH categories if they have multiple CH types.

Supplementary Data 2. Summary of all pairwise CHIP-CHIP enrichment analyses in UK Biobank participants with no prior cancer at baseline (N=453,807). Associations are the result of multivariable logistic regression models adjusted for age, age^2, sex, smoking status, and genetic similarity. Analyses are constructed such that all CHIP mutations are tested as both an exposure and an outcome variable; therefore, each pair is presented twice in the table below. All bolded *P*-values are statistically significant after adjusting for multiple hypotheses ($P \leq 2.7\times10^{-5}$). *P*-values are the result of two-sided tests.

Supplementary Data 3. Summary of all pairwise mCA-mCA enrichment analyses in UK Biobank participants with no prior cancer at baseline (N=453,807). Associations are the result of multivariable logistic regression models adjusted for age, age^2, sex, smoking status, and genetic similarity. Analyses are constructed such that mCAs on each chromosome are tested as both an exposure and an outcome variable; therefore, each pair is presented twice in the table below.
All bolded P-values are statistically significant after adjusting for multiple hypotheses ($P \leq 8.7\times10^{-5}$). P-values are the result of two-sided tests.

Supplementary Data 4. Summary of all pairwise refined mCA-mCA enrichment analyses in UK Biobank participants with no prior cancer at baseline (N=453,807). Associations are the result of multivariable logistic regression models adjusted for age, age^2, sex, smoking status, and genetic similarity. Analyses are constructed such that mCAs defined by chromosomal region and event type are tested as both an exposure and a predictor variable; therefore, each pair is presented twice in the table below. All bolded *P*-values are statistically significant after adjusting for multiple hypotheses ($P \leq 4.9\times10^{-4}$). *P*-values are the result of two-sided tests.

Supplementary Data 5. Summary of CHIP-mCA enrichment analyses in UK Biobank participants with no prior cancer at baseline. Associations are the result of multivariable logistic regression models adjusted for age, age^2, sex, smoking status, and genetic similarity. Analyses are constructed such that CHIP mutations are considered the predictor and mCAs the outcome. All bolded *P*-values are statistically significant after adjusting for multiple hypotheses ($P \leq 4.8\times10^{-5}$). *P*-values are the result of two-sided tests.

Supplementary Data 6. Summary of refined CHIP-mCA enrichment analyses in UK Biobank participants with no prior cancer at baseline. Associations are the result of multivariable logistic regression models adjusted for age, age^2, sex, smoking status, and genetic similarity. Analyses are constructed such that mCAs are defined by chromosomal region and event type. All bolded *P*-values are statistically significant after adjusting for multiple hypotheses ($P \leq 7.6\times10^{-4}$). *P*-values are the result of two-sided tests.

Supplementary Data 7. Summary of all pairwise CHIP-CHIP enrichment analyses in TOPMed participants. Associations are the result of multivariable logistic regression models adjusted for age, age^2, sex, smoking status, genetic similarity, and TOPMed study. Analyses are constructed such that all CHIP mutations are tested as both an exposure and an outcome variable; therefore, each pair is presented twice in the table below. All bolded *P*-values are statistically significant after adjusting for multiple hypotheses ($P \leq 8.7 \times 10^{-5}$). *P*-values are the result of two-sided tests.

Supplementary Data 8. Summary of all pairwise mCA-mCA enrichment analyses in TOPMed participants (N=67,390). Associations are the result of multivariable logistic regression models adjusted for age, age^2, sex, smoking status, genetic similarity, and TOPMed study. Analyses are constructed such that mCAs on each chromosome are tested as both an exposure and an outcome variable; therefore, each pair is presented twice in the table below. All bolded *P*-values are statistically significant after adjusting for multiple hypotheses ($P \leq 8.7 \times 10^{-5}$). *P*-values are the result of two-sided tests.

Supplementary Data 9. Summary of CHIP-mCA enrichment analyses in TOPMed participants. Associations are the result of multivariable logistic regression models adjusted for age, age^2, sex, smoking status, genetic similarity, and TOPMed study. Analyses are constructed such that mCAs are defined by chromosomal region and event type. All bolded *P*-values are statistically significant after adjusting for multiple hypotheses ($P \leq 7.6 \times 10^{-4}$). *P*-values are the result of two-sided tests.

Supplementary Data 10. PLCO CHIP-autosomal mCA co-occurrences. Shaded co-occurrences were enriched in UKBB participants without prior cancer. Values within the table below represent the number of participants with a given co-occurrence.

Supplementary Data 11. Prior cancer cases by cancer group.

Supplementary Data 12. Frequencies of detectable CH in UKBB participants with prior cancer (N=24,634) compared to UK Biobank participants without prior cancer (N=453,807) at baseline. Analyses are the result of multivariable logistic regression adjusted for age, age^2, sex, smoking status, and genetic similarity. All *P*-values are the result of two-sided tests and are bolded if $P < 0.05$.

Supplementary Data 13. Summary of all pairwise CHIP-CHIP enrichment in UK Biobank participants with prior cancer (N=24,634). Associations are the result of multivariable logistic regression models adjusted for age, age^2, sex, smoking status, and genetic similarity. Analyses are constructed such that all CHIP mutations are tested as both an exposure and an outcome variable; therefore, each pair is presented twice in the table below. All bolded *P*-values are statistically significant after adjusting for multiple hypotheses ($P \leq 2.7 \times 10^{-5}$). *P*-values are the result of two-sided tests.

Supplementary Data 14. Summary of all pairwise mCA-mCA enrichment in UK Biobank participants with prior cancer. Associations are the result of multivariable logistic regression models adjusted for age, age^2, sex, smoking status, and genetic similarity. Analyses are

constructed such that all CHIP mutations are tested as both an exposure and an outcome variable; therefore, each pair is presented twice in the table below. All bolded *P*-values are statistically significant after adjusting for multiple hypotheses ($P \leq 8.7 \times 10^{-5}$). *P*-values are the result of two-sided tests.

Supplementary Data 15. Summary of CHIP-mCA enrichment in UK Biobank participants with prior cancer (N=24,634). Associations are the result of multivariable logistic regression models adjusted for age, age^2, sex, smoking status, and genetic similarity. Analyses are constructed such that CHIP mutations are considered the predictor and mCAs the outcome. All bolded *P*-values are statistically significant after adjusting for multiple hypotheses ($P \leq 4.8 \times 10^{-5}$). *P*-values are the result of two-sided tests.

Supplementary Data 16. Random effects meta-analysis of TOPMed and UK Biobank participant characteristics and their association with CH types. Associations are the result of random-effects meta-analyses of TOPMed (N=67,390) and UK Biobank (N=453,807) participants adjusted for age, age^2, sex, smoking status, and genetic similarity. Covariates were not included when they were the primary predictor. All *P*-values are the result of two-sided tests and are bolded if $P < 0.05$.

Supplementary Data 17. Association between CH types, leukocyte telomere length, and 22 hematopoietic-related phenotypes. Associations are the result of multivariable linear regression adjusted for age, age-squared, sex, smoking status, and genetic similarity. All bolded *P*-values are significant after adjustment for multiple hypotheses ($P \leq 3.33 \times 10^{-3}$). *P*-values are the result of two-sided tests. Analyses were performed on 453,807 cancer-free UK Biobank participants. Sample size varies by CH type.

Supplementary Data 18. Risk of myeloid malignancy associated with CH types. Shaded co-occurrences are harbored by ≥ 5 participants with myeloid malignancy and reach a multiple testing threshold of 7.90E-04. Analyses are the result of Cox proportional hazards models adjusted for age, age-squared, sex, smoking status, and genetic similarity. *P*-values are the result of two-sided tests. Estimates that did not converge are not reported. Sensitivity analyses removed participants with abnormal white blood cell or platelet counts. Some estimates no longer converged due to decreased case counts and are therefore reported with NA. Shaded, bolded co-occurrences are harbored by ≥ 5 participants with myeloid malignancy, HR≥40 and *P*-value $\leq 7.9 \times 10^{-4}$.

Supplementary Data 19. Risk of lymphoid malignancy associated with CH types. Shaded co-occurrences are harbored by ≥ 5 participants with lymphoid malignancy and reach a multiple testing threshold of 7.90E-04. Analyses are the result of Cox proportional hazards models adjusted for age, age-squared, sex, smoking status, and genetic similarity. *P*-values are the result of two-sided tests. Estimates that did not converge are not reported. Sensitivity analyses removed participants with abnormal white blood cell or platelet counts. Some estimates no longer converged due to decreased case counts and are therefore reported with NA. Shaded, bolded co-occurrences are harbored by ≥ 5 participants with lymphoid malignancy, HR≥40 and *P*-value $\leq 7.9 \times 10^{-4}$.

Supplementary Data 20. Summary of CHIP genes included in UK Biobank and TOPMed co-occurrence analyses.

Supplementary Data 21. Summary of UK Biobank hematopoietic-related phenotypes.

Supplementary Data 22. ICD-10 codes used to define hematologic malignancy diagnoses.
