# Supplementary material for: Co-occurring clonal hematopoiesis exhibits strong selection and high leukemia risk
Source: Nat Commun. 2026 May 21;17:6682. doi: 10.1038/s41467-026-73302-x (PMC13385913; doi:10.1038/s41467-026-73302-x)
Supplement: Supplementary file 2 — Transparent Peer Review file [file 41467_2026_73302_MOESM2_ESM.pdf]

nature portfolio

Peer Review File

Co-occurring clonal hematopoiesis exhibits strong selection and high leukemia risk

Corresponding Author: Ms Kara Barnao

This manuscript has been previously reviewed at another journal. This document only contains information relating to versions considered at Nature Communications.

This file contains all reviewer reports in order by version, followed by all author rebuttals in order by version.

Version 0:

Reviewer comments:

Reviewer #2

(Remarks to the Author)
The authors have addressed my previous concerns in this revised version.

This is comprehensive study examining CHIP and mCA co-occurrences and its role in hematological malignancies. The work will be a valuable resource to the field and I recommend for publication.

Reviewer #3

(Remarks to the Author)
In this revised manuscript, the authors significantly updated the Discussion to better understand the current results with those from prior studies. They also updated the discussion to better reflect on the limitation of the study.
The authors did not update the co-occurrence figures to incorporate arm-level direction (gain, loss, LOH). The authors stated in their rebuttal that they did so to avoid the stress of multiple testing. While I understand the statistical concern, I strongly believe that from biological point of view, that co-occurrence figure needs to have the information of directionality. Unless the authors have some strong reason to object to this, I highly recommend to do this.

Reviewer #5

(Remarks to the Author)
The authors have addressed all points from the previous reviewer. Aside from the following minor technical corrections, I have no further comments:

Line 536: Remove the date inserted after "chr9."
Methods: Please verify that the R version is indicated throughout the text instead of the RStudio version.

Open Access This Peer Review File is licensed under a Creative Commons Attribution 4.0 International License, which permits use, sharing, adaptation, distribution and reproduction in any medium or format, as long as you give appropriate credit to the original author(s) and the source, provide a link to the Creative Commons license, and indicate if changes were made.
In cases where reviewers are anonymous, credit should be given to 'Anonymous Referee' and the source.
The images or other third party material in this Peer Review File are included in the article’s Creative Commons license, unless indicated otherwise in a credit line to the material. If material is not included in the article’s Creative Commons license and your intended use is not permitted by statutory regulation or exceeds the permitted use, you will need to obtain permission directly from the copyright holder.
To view a copy of this license, visit https://creativecommons.org/licenses/by/4.0/

RESPONSE TO REVIEWERS' COMMENTS

Reviewer #2 (Remarks to the Author):
The authors have addressed my previous concerns in this revised version.
This is comprehensive study examining CHIP and mCA co-occurrences and its role in hematological malignancies. The work will be a valuable resource to the field and I recommend for publication.

We thank the Reviewer for their positive feedback and for recognizing the value of our work.

Reviewer #3 (Remarks to the Author):

In this revised manuscript, the authors significantly updated the Discussion to better understand the current results with those from prior studies. They also updated the discussion to better reflect on the limitation of the study.
The authors did not update the co-occurrence figures to incorporate arm-level direction (gain, loss, LOH). The authors stated in their rebuttal that they did so to avoid the stress of multiple testing. While I understand the statistical concern, I strongly believe that from biological point of view, that co-occurrence figure needs to have the information of directionality. Unless the authors have some strong reason to object to this, I highly recommend to do this.

We thank the Reviewer for this important suggestion. We agree that incorporating chromosome arm-level resolution and distinguishing alteration types (gain, loss, and copy-neutral loss of heterozygosity (CNLOH)) provides additional biological insight into CH co-occurrence patterns.

In response, we performed additional mCA-mCA and CHIP-mCA logistic regression analyses at this resolution. These results are presented in newly added **Supplementary Figures 1 and 2**, included below this response, as well as **Supplementary Data 4 and 6**. These analyses confirm and extend our original findings: in general, effect sizes and statistical significance are strengthened when incorporating alteration type, suggesting that chromosome-level analyses may underestimate the magnitude of specific associations (e.g., *JAK2*-chr9: OR=481.4, 95% CI=[360.6-642.8], $p < 2.2\times10^{-308}$; *JAK2*-chr9pLOH: OR=1741.4, 95% CI=[1261.3-2404.3], $p < 2.2\times10^{-308}$).

We have also added corresponding text in the **Results** section (Lines 231-236, 264-267, and 273-276) to highlight these findings. While we retain the chromosome-level figures in the main text, the new analyses are included to address this important biological consideration.

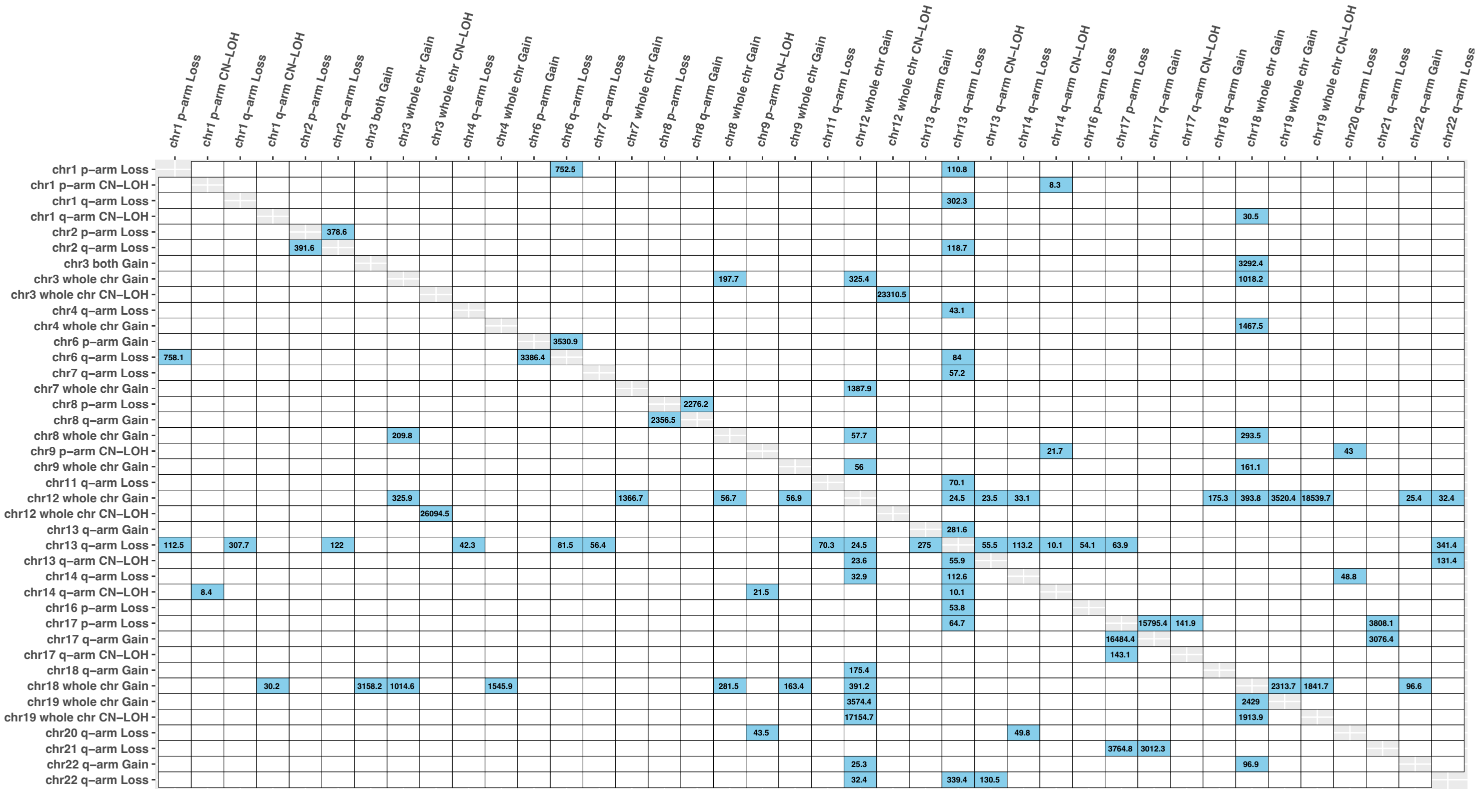

Supplementary Fig. 1. Refined analyses of enriched mCA co-occurrence in UK Biobank participants without prior cancer (N=453,807). Co-occurrence of autosomal mCAs defined by chromosomal region and event type. Associations were tested using multivariable logistic regression adjusted for age, age^2, sex, smoking status (never, former, current) and genetic similarity; odds ratios (ORs) are shown for associations with $P \leq 0.05$ (two-sided tests). Colored cells indicate Bonferroni-corrected significance ($P \leq 4.9 \times 10^{-4}$).

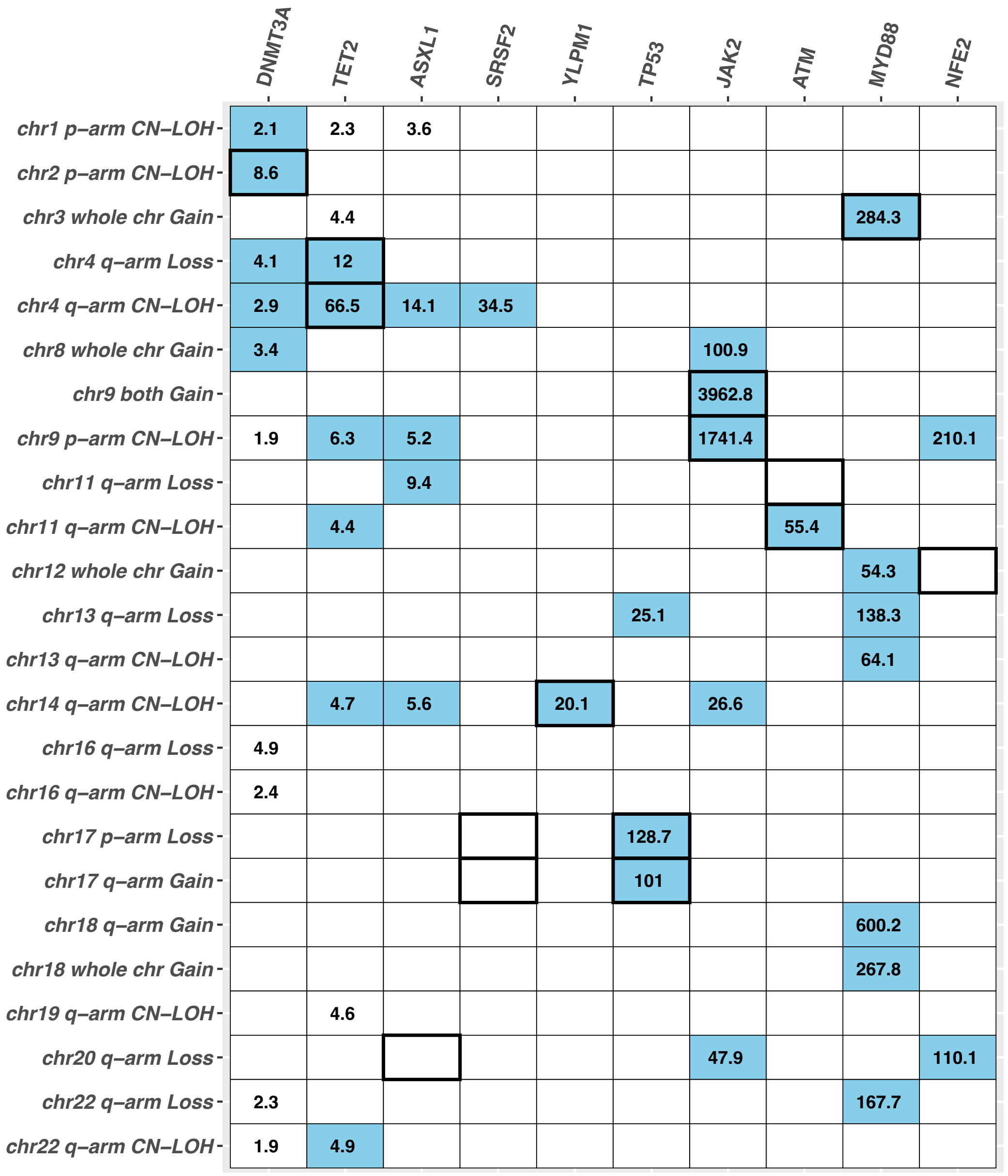

Supplementary Fig. 2. Refined analyses of enriched CHIP-mCA co-occurrence in UK Biobank participants without prior cancer (N=453,807). Co-occurrence of CHIP mutations and autosomal mCAs defined by chromosomal region and event type. Associations were tested using multivariable logistic regression adjusted for age, age^2, sex, smoking status (never, former, current) and genetic similarity; odds ratios (ORs) are shown for associations with $P \leq 0.05$ (two-sided tests). Colored cells indicate Bonferroni-corrected significance ($P \leq 7.6 \times 10^{-4}$). Bold boxes indicate the chromosomal location of each CHIP gene.

Reviewer #5 (Remarks to the Author):

The authors have addressed all points from the previous reviewer. Aside from the following minor technical corrections, I have no further comments:

Line 536: Remove the date inserted after "chr9."
Methods: Please verify that the R version is indicated throughout the text instead of the RStudio version.

We thank the Reviewer for identifying these minor issues. Regarding line 536, we did not intend for a date to be inserted after chr9; rather, there should be two citations of prior work related to *NFE2*-chr9: "*JAK2*-chr14, *JAK2*-chr20, *NFE2*-chr9[22,23]". We believe the date was inserted when the text was converted to PDF. This has been updated in the manuscript.

We also carefully reviewed the Methods section and ensured that the R version, as opposed to the RStudio version, is consistently reported throughout the manuscript.
